# On the stability of norms and norm-following propensity: a cross-cultural panel study with adolescents

**DOI:** 10.1007/s10683-024-09821-5

**Published:** 2024-05-06

**Authors:** Erik O. Kimbrough, Erin L. Krupka, Rajnish Kumar, Jennifer M. Murray, Abhijit Ramalingam, Sharon Sánchez-Franco, Olga L. Sarmiento, Frank Kee, Ruth F. Hunter

**Affiliations:** 1 Smith Institute for Political Economy and Philosophy, Argyros College of Business and Economics, Chapman University, Orange, USA; 2 School of Information, University of Michigan, Ann Arbor, USA; 3 Department of Economics, Queen’s Business School, Queen’s University Belfast, Belfast, UK; 4 Centre for Public Health, Queen’s University Belfast, Belfast, UK; 5 Department of Economics, Appalachian State University, Boone, USA; 6 School of Medicine, Universidad de los Andes, Bogotá, Colombia

**Keywords:** Norms, Experimental economics, Heterogeneity, C93, D01, D91

## Abstract

Norm-based accounts of social behavior in economics typically reflect tradeoffs between maximization of own consumption utility and conformity to social norms. Theories of norm-following tend to assume that there exists a single, stable, commonly known injunctive social norm for a given choice setting and that each person has a stable propensity to follow social norms. We collect panel data on 1468 participants aged 11–15 years in Belfast, Northern Ireland and Bogotá, Colombia in which we measure norms for the dictator game and norm-following propensity twice at 10 weeks apart. We test these basic assumptions and find that norm-following propensity is stable, on average, but reported norms show evidence of change. We find that individual-level variation in reported norms between people and within people across time has interpretable structure using a series of latent transition analyses (LTA) which extend latent class models to a panel setting. The best fitting model includes five latent classes corresponding to five sets of normative beliefs that can be interpreted in terms of what respondents view as “appropriate” (e.g. equality vs. generosity) and how they view deviations (e.g. deontological vs. consequentialist). We also show that a major predictor of changing latent classes over time comes from dissimilarity to others in one’s network. Our application of LTA demonstrates how researchers can engage with heterogeneity in normative perceptions by identifying latent classes of beliefs and deepening understanding of the extent to which norms are shared, stable, and can be predicted to change. Finally, we contribute to the nascent experimental literature on the economic behavior of children and adolescents.

**Supplementary Information:**

The online version contains supplementary material available at 10.1007/s10683-024-09821-5.

## Introduction

Economists have come to understand that social behavior is, among other things, norm-governed. Norms are often conceptualized as beliefs about and standards of appropriate behavior (Bicchieri, 2006; Cialdini & Trost, 1998); they also coordinate actions and expectations in interactions with multiple equilibria (Binmore & Samuelson, 2006; Gintis, 2009; Sugden, 1995).^1^ Models of norm-driven choice assume that people seek both their own consumption utility and to adhere to commonly known injunctive norms, creating trade-offs when those two objectives conflict, which individuals resolve differently depending on the weight they assign to normative goals.^2^ Thus, to understand how norms shape decisions, both the norms themselves and the individual propensity to follow them have become objects of study and quantification (Kimbrough & Vostroknutov, 2016, 2018; Krupka & Weber, 2013).

With measures of norms and/or norm-following propensity in hand, researchers attempt to assess the predictive validity of these models (see e.g., Kranton 2002, 2005, Benjamin, Choi, and Strickland 2010, Chang et al., 2019, Eckel et al., 2021, Kimbrough & Vostroknutov, 2016, and Krupka & Weber, 2013). This approach depends crucially on a few assumptions that have not been widely tested empirically. First, these models and associated measurement strategies assume what we call “norm uniqueness”, i.e. that reports about norms capture a single shared norm, with error, and that the error is mere noise. Second, they assume “norm stability”, or that absent intervention the norm doesn’t change, such that the average change in reports about the norm should equal zero in a panel setting. Finally, these theories assume “preference stability”: that individual norm-following propensity is a constant individual-level characteristic.

In this paper, we elicit norms and a proxy measure for individual norm-following propensity in two periods using an experiment to test these assumptions (Kimbrough & Vostroknutov, 2016, 2018; Krupka & Weber, 2013).^3^ Specifically, we exploit a convenience panel with a large sample of adolescents: 1,468 student participants aged 11–15 years who were subjects in a study on smoking norms and social networks.^4^ As a control question, the study collected panel data on normative beliefs about dictator game giving and a proxy measure of propensity to follow norms two times separated by 10 weeks. Since the dictator game is a workhorse in the norms literature, this presented an opportunity to explore these important conceptual and methodological issues. Collecting such data among adolescents also presents a valuable opportunity since moral reasoning and social cognitive skills are both developing in these years (Maggian & Velleval, 2016; Sutter et al., 2019). We use these data to test the hypotheses that norms and norm-following propensity are stable, on average, at the population level, and then at the individual level.

We find that norm-following propensity is stable, on average, but reported norms show some evidence of change, even over a 10-week period. While we did not start our research project expecting to find unstable norms, that is what our data reveal. Thus, we take the opportunity to explore the nature of this instability. Since peoples’ normative views of the “same game” seem to be changing over time, this raises the question of whether people perceive the same norm to begin with (i.e. evidence of instability may also contain evidence of non-uniqueness). Thus, we explore whether individual-level variation in reported norms between people and within people across time has interpretable structure using latent transition analyses, which extend latent class models to a panel setting. Our analysis reveals that individual-level variation in reported norms has structure. The best fitting model includes five latent classes corresponding to five sets of normative beliefs that can be interpreted in terms of what respondents view as “appropriate” (e.g. equality vs. generosity) and how they view deviations from the most appropriate action (e.g. deontological vs. consequentialist). We also find that many subjects appear to change latent classes over time. Thus, subjects arrive at our study with heterogeneous views about what is normatively appropriate in the dictator game, and many exhibit some change in those views over time.

This raises the question of why normative views are changing over our 10-week period in the absence of intervention. A reasonable hypothesis is that a subject pool in which peers repeatedly interact between waves should only get better at guessing each other’s views in the presence of incentives to coordinate. With initial heterogeneity in beliefs, the incentive to coordinate may therefore encourage subjects to report entirely different normative beliefs at wave 2 if they learn that their beliefs differ from their peers. Thus, we exploit another convenient feature of our data, which includes measures of peer networks, and we show that observed changes in normative perspective are not arbitrary but can be predicted by information about subjects’ similarity to their peer networks.^5^ We find that subjects who are more dissimilar to their peers are more likely to be categorized in a different latent class in wave 2. Surprisingly this does not lead to a higher coordination rate in wave 2. Thus, we can predict to some extent who changes, but not how they will change. This suggests that there is genuine normative uncertainty or disagreement in this environment. More broadly, this exercise serves as a proof of concept that illustrates the kinds of questions researchers can ask when analyzing heterogeneous normative beliefs.

Our first contribution is to advance research on norms by testing some of the basic assumptions of models of norm-driven behavior. We provide evidence that the propensity to follow norms is stable, on average, in our sample. We show that normative beliefs are not entirely consistent over time, on average, and we thus unpack the working assumption that there is a single, stable injunctive norm in any choice context. We argue that researchers should be careful to conceptualize injunctive norms as a complete profile of beliefs about the *relative* appropriateness of all possible actions. We show that such normative belief profiles, even in the dictator game, are strikingly heterogeneous and that this heterogeneity can be decomposed into meaningful classes. In this, we join a recent paper by Fromell et al., (2021) which also finds that a plurality of norms exists in the specific context of norms that regulate the trade-off between wealth accumulation through saving and sharing income with kin and neighbors in rural Kenya.

Our second contribution is methodological; we use repeated experimental elicitation and latent transition analysis to make sense of observed heterogeneity in our data. The novel approach using latent transition analysis allows us to identify the relevant classes of normative beliefs and assess the extent to which subjects change classes over time. Latent variable models allow us to identify distinct “types” and transitions between them in a constant decision environment. Using adolescents has some drawbacks related to how malleable and sensitive to social influences they may be. However, it also has some strengths for the question we are looking at and the methods we employ. Notably, this subject pool is brought together daily in a social context and in a similar way each day (i.e., they attend school). The attrition in our subject pool is thus minimal. In addition, the students are motivated to participate and interested in being part of the study. As such, their attention is likely high. Taken together, these factors mean that our subject pool is ideal for demonstrating the promise of latent transition analysis, which requires a large panel with minimal attrition.

Third, we show how to use the identified classes to characterize and predict normative belief change such that people who hold similar normative views to their peers, are less likely to change their views. With these advances in how we treat variation, we hope researchers will have much more to say about when a norm is shared in a population, whether it is strong or weak, and how and at what moment norm change (at the individual and aggregate level) has taken place.

Finally, we contribute to the nascent experimental literature on the economic behavior of children and adolescents. In particular, we contribute to a topic that has received little attention—the study of coordination games among this population. That said, the malleability of opinion or belief formation at this age suggests that our findings could be an upper bound on norm heterogeneity, norm profiles and norm change.

## Background and motivation

We start from the premise that choice data is not sufficient to reveal an injunctive norm, because the same choice can be attributable to different motives (e.g. an equal split in the dictator game could reflect both a norm of generosity and a norm of egalitarianism). Thus, economists have developed methods designed to elicit normative beliefs directly, and the idea of norm uniqueness has guided the design of these measurement techniques (Nosenzo & Görges, 2020). Krupka and Weber (2013) elicit individual normative beliefs by asking participants to play a “pure matching” coordination game (Mehta et al., 1994; Schelling, 1960) in which their goal is to anticipate the extent to which others in their group will rate an action as socially appropriate or inappropriate, and to respond accordingly. The incentives do not reward participants for revealing their own views, but instead reward them for matching their appropriateness ratings with other participants in the experiment. This technique neatly captures the idea of a norm as a set of shared beliefs about what is and is not appropriate, and reported norms have been shown to be robust to alternative (non-normative) focal points (Fallucchi & Nosenzo, 2021).

Norm uniqueness also infuses how norms are discussed in the literature; the typical approach describes a norm in terms of a single prescriptive action (e.g. the “norm is to tip 20%” or “to split 50–50”), with all remaining actions (implicitly) seen as equally inappropriate actions that should not be taken (e.g., Akerlof & Kranton, 2005; Andreoni & Bernheim, 2009). This has influenced the interpretation of data collected to measure norms: *the* norm is often identified by the action that was given the highest mean (or modal) normative evaluation by participants (e.g. “there is a clear norm of equal division” in the dictator game, Kimbrough & Vostroknutov, 2016, p. 633).

However, as several papers note, using only the most appropriate action to capture the norm discards valuable information about normative beliefs (Chang et al., 2019; Krupka & Weber, 2013). Instead, thinking of norms as a profile of normative beliefs across a set of related actions emphasizes that norms define both what is most appropriate and how bad it is to deviate from that (Nosenzo & Görges, 2020). The profile of normative evaluations across a set of related actions conveys both of those features of a norm. That is, the *shape* of the function mapping actions to evaluations of appropriateness is what really defines a norm. The relevant normative tradeoff is then characterized by a norm-dependent utility function that incorporates heterogeneity in norm-following propensity (Bicchieri, 2006; Cappelen et al., 2007; Kessler & Leider, 2012; Kimbrough & Vostroknutov, 2016, 2022; Krupka & Weber, 2013; Lopez-Perez, 2008).^6^

Even when researchers have focused on the profile of responses in their analysis, the emphasis on shared-ness has led to a working assumption that there is a single norm profile representing the injunctive norm in a given choice context. One exception is a recent paper by Fromell et al., (2021). They use a lab-in-the-field experiment in Kenya and identify multiple parallel social norms that regulate the trade-off between wealth accumulation through saving and sharing income with kin and neighbors. Specifically, they find that one group (a minority) of participants perceive a “strict” norm of sharing while a second group (most participants) recognize moderate accumulation of wealth as socially acceptable and yet a third group (about a quarter) of participants perceive a “pro-saving” norm, whereby keeping most of one’s wealth for oneself is the most appropriate course of action. Notwithstanding the Fromell et al., paper, it is a wide-spread practice to assume that individual deviations from the average (or sometimes modal) response for any one action being evaluated represent measurement error (Krupka & Weber, 2013). But if individual deviations from the average norm profile have structure, then they are not “errors”. Rather, they may instead be evidence of multiple contemporaneous norms and/or ongoing normative change. These two possibilities are the focus of this paper.

There is some cause for skepticism about the assumption that variation reflects mere measurement error. One reason is that norms are arguably indexed to identity groups such that normative prescriptions depend on one’s identities and their salience at a particular moment (Akerlof & Kranton, 2000). Consistent with this view, Burks and Krupka (2012), Chang et al., (2019), Pickup et al., (2021), and Groenendyk et al., (2021) provide evidence that different social groups, such as managers and employees, democrats and republicans, or liberals and conservatives, disagree about the appropriateness of a variety of actions. More important for the purposes of this paper, starting even before the most widely used techniques for measuring norms were published, evidence began to accumulate that different people sometimes apply different norms to the same experimental situation (e.g. Yaari and Bar-Hillel (1984), and later Rueben and Riedl (2013) and Carpenter and Matthews (2008)). Thus, there is evidence of normative heterogeneity among anonymous participants interacting in the sparse, abstract contexts studied in the lab, even absent cues about identity.

Another working assumption in the literature is that norms are stable over time in the absence of interventions that alter peoples’ incentives or information about a given setting. Without panel data, it is impossible to test stability directly. Most research that addresses norms over time tests whether a change has been induced by an intervention rather than testing their temporal stability (see for example, Chang et al., 2019). Moreover, research on the robustness or stability of the results produced by norm-elicitation techniques has thus far largely focused on the robustness of the mean, e.g. asking whether the mean report is influenced by the perspective from which the task is described (1st, 2nd or 3rd person) (see e.g. Erkut et al., 2015). Alternatively, it has focused on whether the norm is robust to whether the elicitation is conducted with the same participants whose choices are observed or instead conducted with a separate sample drawn from the same participant pool (D’Adda et al., 2016). Further, all of these studies employ adult populations while we are focusing on stability among a younger group who, there is some evidence to suggest, may be more sensitive to norms or for whom norms may be less stable than for adult populations (Blakemore & Mills, 2014; Do et al., 2020). In particular, there is evidence that peer effects play an especially important role in shaping the behavior of children (see e.g. List et al., 2023).

There is strong evidence to suggest that, on average, elicited norms for the dictator game do not differ much among adult (primarily WEIRD) populations (D’Adda et al., 2016; Erkut et al., 2015; Kimbrough & Vostroknutov, 2018), and they appear to be fairly well-established by the time children become adolescents. Sutter et al., (2019) and List et al., (2023) review economic behavior of children and adolescents and find that by the time children enter school (around age 6), rationality and social preferences for fairness are fairly stable.^7^ In particular, they note that “…fairness concerns seem deep rooted and [are] early developed.” (Sutter et al., p. 113; Blake et al., 2015; Fehr et al., 2008). A smaller group of papers show that even children as young as 3–5 years old who understand the emotional consequences of moral violations (they feel bad and another person might feel bad) allocate stickers more generously in the dictator game. Maggian and Villeval (2016) show that the majority of adolescents (ages 7–14) who could lie for advantage, do not do so. Both of these studies suggest that the ability to understand and incorporate norms into decision making is present in the population we study. Evidence on peer effects noted above suggests that children regularly incorporate social information into their decisions, but, to our knowledge, there has been little work directly measuring normative beliefs among children. Our research thus contributes to this literature. Moreover, Grueneisen et al., (2015a, 2015b) and Grueneisen et al., (2015b) show that from at least the age of 5 on, children can coordinate with peers by converging on a salient solution, and Brocas and Carillo (2021) show that children become better at coordinating as they become adolescents. This suggests that adolescents are also likely to be capable of playing our coordination game, which we use to measure normative beliefs.

A final assumption undergirding models of norm-dependent decision-making is “preference stability”. In the tradition of Stigler and Becker (1977), economists tend to treat preference parameters as exogenous. Norm-dependent utility is intended to explain context-dependent behavior by reference to context-dependent norms, which enter the utility of agents who care about following them to varying degrees. For example, agents are assumed to maximize norm-dependent utility ${v}_{i}\left(x\right)={u}_{i}\left(x\right)+{\phi }_{i}\eta (x)$, where ${u}_{i}(x)$ is *i*'s consumption utility at outcome $x$, $\eta \left(x\right)\in [-\mathrm{1,1}]$ is the normative appropriateness of outcome $x$ according to the context-dependent norm, and ${\phi }_{i}$ is the weight placed on norm-following by *i*. Kimbrough and Vostroknutov (2016, 2018) introduced a method for eliciting a proxy for the parameter ${\phi }_{i}$, but to our knowledge, no one has assessed the test–retest reliability of this measure.

In sum, in the absence of intervention, the literature on norms makes assumptions that we call “norm uniqueness”, “norm stability”, and “preference stability”. To our knowledge, these assumptions have not been thoroughly tested, and so we set out to do so. Ultimately, we reject the first two hypotheses. We show that there is significant heterogeneity in perceptions of the norm that are not obvious when only looking at an aggregate norm measurement. We also show that perceptions of norms enjoy some stability over time, but that a major predictor of change in normative perception comes from dissimilarity to others in one’s network. Perhaps most importantly, we demonstrate how researchers might engage with this heterogeneity in normative perceptions by identifying classes of norm perceptions (e.g. a deontological equality norm or a consequentialist generosity norm) and asking how those classes can be used to deepen our understanding of norm emergence and norm change.

## Methods

As part of the MECHANISMS study (Hunter et al., 2020), we collected repeated measures data on 1468 students aged 11–15 years old in 15 schools in and around Belfast, Northern Ireland and in Bogotá, Colombia. Participation was open to all students in a school-year group (approximately 100 students per school); uptake was approximately 90% at each location. These data provide us with two measures from each participant of a proxy for norm-following propensity and beliefs about injunctive norms in the dictator game, collected approximately 10 weeks apart.

These measures were not expected to change between waves as they were collected as controls alongside a broad set of other measures including norms related to smoking/vaping, self-reports of smoking behavior and intentions, social networks, and personality traits. These control measures served two purposes. First, since the dictator game is the most widely studied game in the social norms literature and yields remarkably consistent responses, on average, in norm-elicitation experiments, we had strong priors about what the elicited norm (hereafter DGN) would look like, on average.^8^ Thus, deviations from this prior would serve as a sort of warning light to us regarding participants’ understanding of the norm elicitation procedure which was also being used to measure norms related to smoking. Second, and more important for the purposes of this paper, while we anticipated that the interventions would influence norms related to smoking–since they were designed to do so–we had no reason to expect the interventions to influence the DGN or in norm-following propensity since the interventions were not designed to influence either of these. Thus, our (null) hypotheses were that neither the DGN nor the norm-following propensity would change, on average over time.

In this paper, we focus only on the measures of dictator game norms, norm-following propensity, and social networks. Data about pre-treatment norms and behavior related to smoking and on the effects of our anti-smoking interventions are reported in Murray et al., (2021). All instruments were previously translated and adapted to Spanish. Data collection was conducted on individual tablet computers using Qualtrics. Instructions were read aloud by a monitor as participants followed along on screen. Screenshots of the interface and the instructions in English are reported in Appendix B. Participants received no feedback about their choices or the choices of others until the completion of the second survey wave. Participants were paid for either wave 1 or wave 2 for each task with equal probability. All payments were delivered, in cash in Northern Ireland and in a gift card in Bogotá, at the conclusion of the MECHANISMS study.

### Dictator game norms

Following the protocol developed by Krupka and Weber (2013) we measured participants’ normative expectations in the dictator game using an incentivized coordination game. Participants read the following vignette in Northern Ireland; these instructions were translated into Spanish and adjusted for purchasing power parity and instructions compressibility in Bogotá:

*“Individual A and Individual B from the class are randomly paired with each other. Individual A received £10.00. Individual A will then have the opportunity to give any amount of his or her money to Individual B. For instance, Individual A may decide to give £0.00 to Individual B and keep £10.00 for him or herself. Or Individual A may decide to give £10.00 to Individual B and keep £0.00 for him or herself. Individual A may also choose to give any other amount between £0.00 and £10.00 to Individual B. This choice will determine how much money each will receive, privately and in cash, at the end of the experiment.”*

Then, participants were provided with a list of 11 possible actions that a dictator could take (from keeping the entire endowment to giving the entire endowment to the recipient) and asked to report on a 6-point Likert scale whether each action was: ‘extremely socially inappropriate’, ‘very socially inappropriate’, ‘somewhat socially inappropriate’, ‘somewhat socially appropriate’, ‘very socially appropriate’, or ‘extremely socially appropriate’. This task was included in both the pre- and post-intervention survey.

Respondents were told that at the end of the study, we would randomly select one of their two surveys, and then choose one of the possible actions at random to determine their payment. They were told that if their normative evaluation of the chosen action matched the modal response of others in their school-year group, they would receive £10 (10.000 COP in Bogotá); otherwise, they would receive £0 for this task. This incentivizes participants to report shared beliefs about the appropriateness of each action, which is the definition of an injunctive social norm. As in Krupka and Weber (2013), the idea is that the social norm is a focal point that resolves the coordination problem.

### Norm-following propensity

Following the protocol developed by Kimbrough and Vostroknutov (2018), we measured a proxy for norm-following propensity using a variant of the rule-following task (Kimbrough & Vostroknutov, 2016) designed to be culturally portable. Participants are given 50 virtual balls and shown two virtual buckets, one yellow and one blue.

Participants are told that for each ball they drag into the yellow bucket, they will earn 10 pence (in Northern Ireland; 200 COP in Bogotá), and for each ball they drag into the blue bucket, they will earn 5 pence (in Northern Ireland; 100 COP in Bogotá). The instructions then state *“The rule is to put the balls in the blue bucket.”* However, there are no costs imposed for violating the experimenter-stated rule, and so following the rule only results in forgoing the opportunity to earn a higher payoff. Participants’ willingness to incur such costs has been shown to correlate with norm-consistent behavior in a variety of tasks that have been used to study social preferences (Kimbrough & Vostroknutov, 2016, 2018; Ridinger, 2018; Thomsson & Vostroknutov, 2017). Participants are told that we will randomly choose either their first or second survey to be the one that counts for payment, and they receive an amount equal to the sum of the value of balls placed in the two buckets. They were also paid based on their decisions from two other incentivized tasks, and they earned an average of £15.52 (31,140 COP in Bogotá). To this we added a base participation payment of £5.00 (5,000 COP in Bogotá).

### Peer networks

In addition to behavioral data, the study also conducted a survey to measure peer networks. These data allow us to assess whether peer effects can explain any observed changes in norms across the survey waves. Participants were asked to nominate up to 10 of their school-year-group peers as friends. We match the listed names to a master list of students in a participant’s year-group. We define the peer network as all those people who a participant nominated as a friend in the social network survey. For comparison, we also look at peer effects from (1) the people in the same classroom as a participant, and, because this was the relevant matching group for the coordination game, (2) the people in the same school-year group as a participant.

## Results: aggregate analysis

The full study sample is described in detail in Murray et al., (2021). Table 1 presents summary statistics of some of the demographic characteristics of our sample. The study was conducted in multiple schools (7 in Northern Ireland and 8 in Bogotá), and each school was treated with either the ASSIST or Dead Cool anti-smoking intervention (8 received ASSIST and 7 received Dead Cool).^9^ Each school consisted of a different number of classes (36 in Northern Ireland and 32 in Bogotá).

**Table 1:** Baseline mean sample characteristics for MECHANISMS schools

	Northern Ireland (N = 7)	Bogotá (N = 8)	All schools (N = 15)
ASSIST (intervention)	4	4	8
Dead Cool (intervention)	3	4	7
No. of classes, N	36	32	68
No. of pupils, n	825	999	1824
Participation, n (%)	764 (92.6%)	892 (89.3%)	1656 (90.8%)
Boys	335 (47.8%)	436 (50.0%)	771 (49.0%)
Girls	355 (50.6%)	431 (49.4%)	786 (50.0%)
Prefer not to say	11 (1.6%)	5 (0.6%)	16 (1.0%)
Age, n (%)			
11 years old	1 (0.1%)	26 (3.0%)	27 (1.7%)
12 years old	279 (39.8%)	320 (36.3%)	599 (37.8%)
13 years old	414 (59.1%)	313 (35.5%)	727 (45.9%)
14 years old	7 (1.0%)	146 (16.6%)	153 (9.7%)
15 or more years old	-	77 (8.7%)	77 (4.9%)

We have a very high participation rate in our studies due to recruitment of whole school year groups. In Northern Ireland the participation rate is 92.6% and in Bogotá it is 89.3%. We have a somewhat larger number of participants from Bogotá than from Northern Ireland (55 vs. 45% of our sample). In both locations, boys and girls each made up about 50% of all participants. Most participants are 12 or 13 years old.

**Fig. 1:**
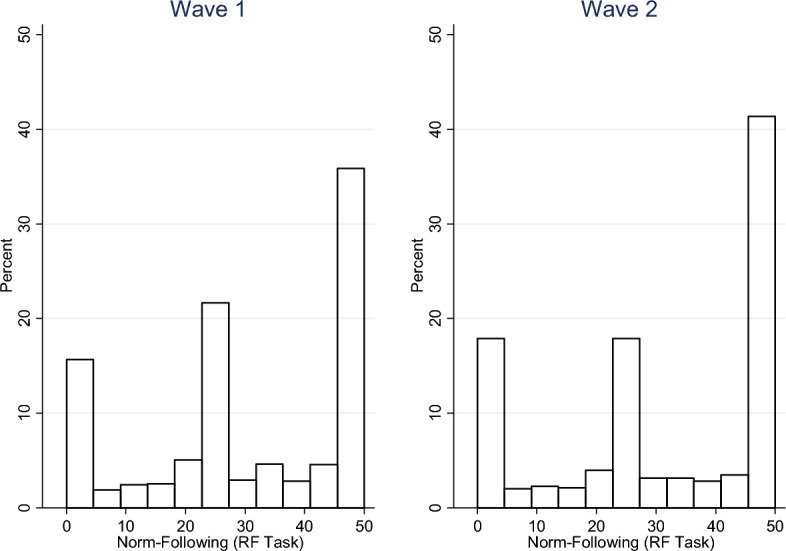
Estimates of norm-following propensity

In what follows, we combine data from both countries since prior empirical work done with adult populations offers no *ex-ante* reason to expect different dictator game norms or norm-following propensities in urban populations (e.g. Kimbrough & Vostroknutov, 2018). Appendix A presents the same analysis for the two settings separately and shows that that are no economically meaningful differences. Figure 1 presents the distribution of the number of balls placed in the blue bucket (i.e., the extent to which people followed the rule) in the RF task in wave 1 and wave 2, which is our proxy for a subject’s norm-following propensity. The Figure shows that there are three main types of behavior when it comes to norm-following: (i) complete disregard for the rule captured by those who put no balls in the blue bucket, (ii) equal split captured by those who allocate half the balls to the blue bucket and half to the yellow bucket, and (iii) complete rule-following captured by those who allocate all 50 balls to the blue bucket. While there are participants who allocate other numbers of balls to the blue bucket in both waves, such allocations are rare and are never more than 5% of participants.

The distribution of RF task behavior appears relatively stable over the two waves. The percentage of those who completely disregarded the is 12% in wave 1 and 15% in wave 2. The percentage of participants with exactly equal splits is just over 14% in wave 1 and slightly under 13% in wave 2. There is a more pronounced increase in complete rule-following from wave 1 (~ 31%) to wave 2 (~ 36%). There is no discernible difference in the percent of participants allocating other numbers of balls to the blue bucket between the two waves.

**Fig. 2:**
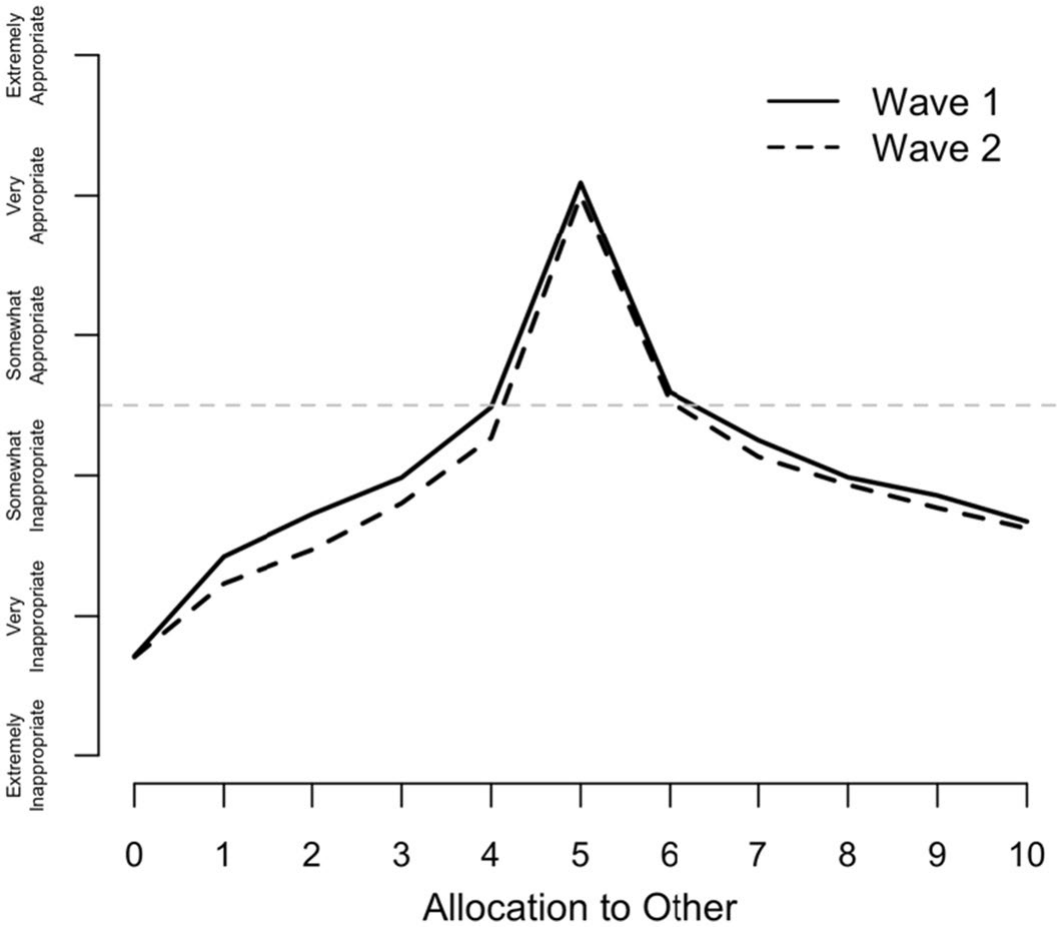
Dictator game norms, on average

Figure 2 shows the profile generated by the average appropriateness rating across all schools for each possible allocation to the recipient in the dictator game in the two waves. On the y-axis the average ratings range from -1 (extremely socially inappropriate) to 1 (extremely socially appropriate). The x-axis records the possible transfer amounts from the dictator to the recipient (ranging between giving nothing, 0, and transferring everything, 10).

Ratings in both waves show very similar patterns. We see that an equal split between the dictator and recipient was viewed as “very socially appropriate” on average, and that there is a sharp decline in the appropriateness ratings for any deviation (on either side) from the equal split. In both waves there is a more gradual decline in appropriateness with increasing distance from the equal split, and allocating 0 to the recipient is viewed as less appropriate than allocating 100% to the recipient. Overall, average norms are fairly stable over time. Except for the focal allocations of 0, 50 and 100% to the recipient, appropriateness ratings are slightly lower in wave 2 than in wave 1.

Table 2 presents summary statistics of the change in rule-following and the change in appropriateness ratings for all the possible dictator allocations between the two waves for all individuals who participated in both waves (N = 1,468). In addition, the Table presents results from Wilcoxon signed rank tests for zero difference between the two waves.^10^

**Table 2:** Change in norm-following and norms

	Measure	Mean	Standard error	Confidence interval	
Norm-following	RF Task	-0.01	0.01	-0.01	0.03
	Give 0	-0.00	0.02	-0.03	0.03
	Give 1	-0.09***	0.02	-0.12	-0.06
	Give 2	-0.10***	0.02	-0.13	-0.07
	Give 3	-0.07***	0.02	-0.10	-0.04
Appropriateness ratings	Give 4	-0.07***	0.02	-0.11	-0.04
	Give 5	-0.04	0.02	-0.07	0.00
	Give 6	-0.03	0.02	-0.07	0.00
	Give 7	-0.06**	0.02	-0.09	-0.02
	Give 8	-0.03	0.02	-0.07	0.00
	Give 9	-0.05	0.02	-0.08	-0.01
	Give 10	-0.03	0.02	-0.08	0.01

^***^ *p* < 0.01, ** *p* < 0.05, * *p* < 0.10, Wilcoxon signed-rank tests with Holm-Bonferroni correction to account for multiple comparisons. N = 1468

The tests in Table 2 confirm earlier impressions from Figs. 1 and 2. The average change in rule-following (proportion of balls in the blue bucket) is small (0.01), and this is not statistically significant. Allocations giving small amounts to the recipient (£1—£4) were rated as significantly less appropriate in wave 2 than in wave 1. However, allocations higher than 50% are not rated significantly differently between waves, with the exception of allocating £7 (at the 5% level). However, an allocation of £7 is seen as *less* appropriate in wave 2.

Importantly, the evaluations of the most salient actions (give 0 and give 5) do not change significantly on average. For the evaluations that do change, the maximum average change is approximately -0.1, which is equivalent to ¼ of the change that would occur if participants all changed their evaluation by a single category (e.g. from “somewhat socially inappropriate” to “very socially inappropriate”). Compare this to Chang et al., (2019) Tables A.1 and A.4, in which an experimental treatment causes normative evaluations of dictator game decisions to change by approximately 0.5 (5 × as much) in a between-participant design. Thus, though the within-participant differences are statistically significant, they are not large on average, and the overall visual profile of the average normative beliefs remains quite consistent.

**Fig. 3:**
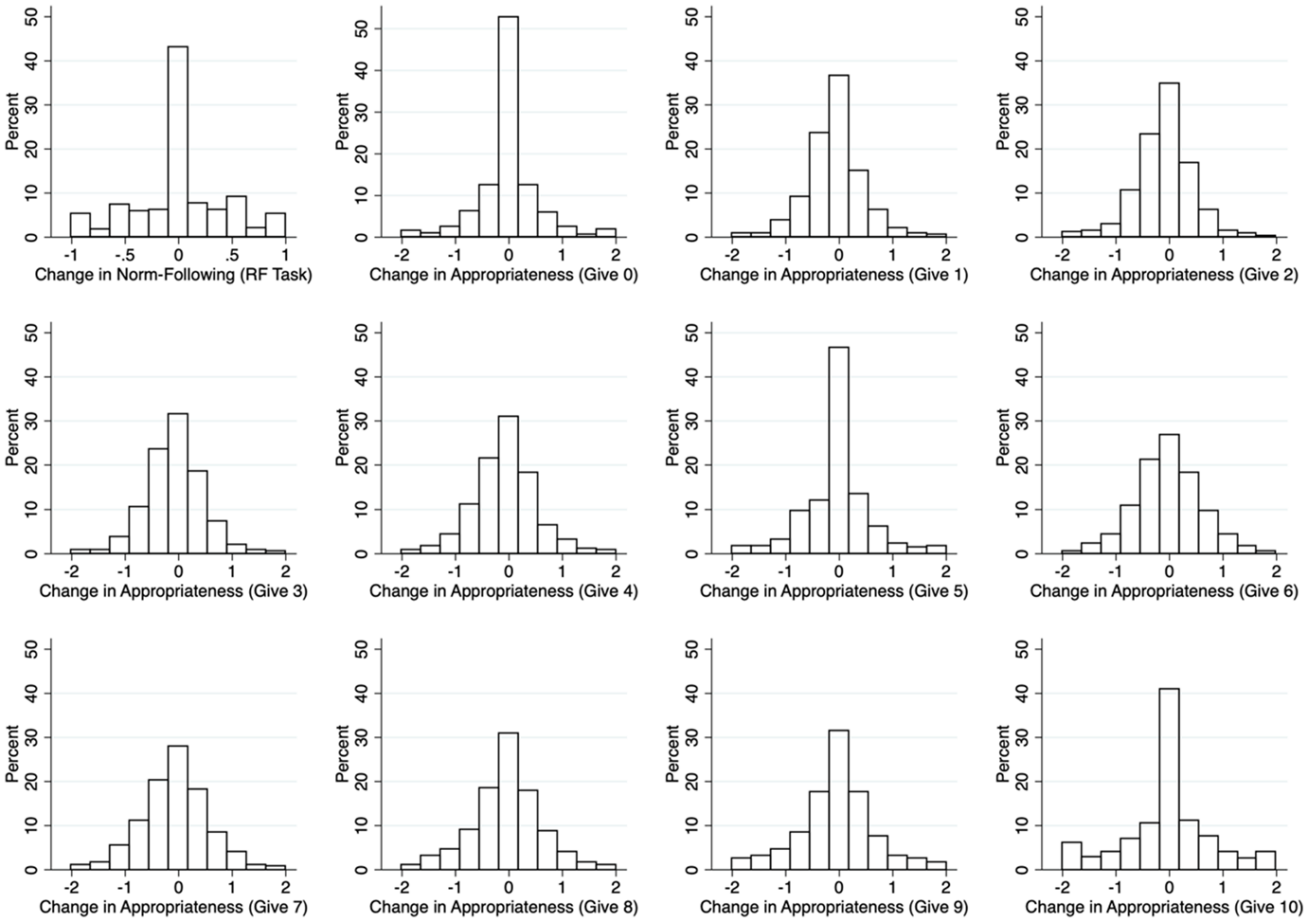
Histograms of individual-level changes in norms and norm-following

Table 2 suggests that average changes are small overall. This could result from small changes at the individual level, or from individual changes in opposite directions that cancel each other out. To investigate this issue, Fig. 3 plots the distributions of all individual changes in norm-following (top row, first panel) and changes in appropriateness ratings of each potential dictator allocation.

In the top left panel of Fig. 3, we see that a material proportion of participants (> 40%) do not change their behavior in the rule-following task. No more than 10% of the participants change their choices completely between waves (e.g. moving from fully rule-following to fully rule-breaking or vice versa). Moreover, the magnitudes of changes are small on average.

The remaining panels depict histograms of the appropriateness ratings for each action (e.g. “give 0”). Visually, we find little evidence of substantial changes in ratings from wave 1 to wave 2 for transfers of 0, £5 or £10. In particular, more than 50%, 45% and 40% of participants respectively do not change their ratings between waves. For all other allocation levels, the modal change between waves is still zero. Even when there are changes by participants, these changes are small. In all eleven possible dictator allocations, more than 70% of participants either do not change their rating or the change is a single appropriateness category.

These findings suggest that both norm-following and norms on dictator allocations are, on average, relatively stable over the 10-week period in our study. However, some of the changes in normative beliefs are statistically significant, and we do note that there are changes at the individual level. The rest of the paper explores the sources of this individual heterogeneity and asks to what extent it reflects the existence of multiple contemporaneous norms and of ongoing normative change.

### Results: evidence of multiple norms

While the between-participant average normative valences across the eleven possible actions in the dictator game generate the singular, familiar pattern shown in Fig. 2, the averages conceal substantial heterogeneity in the patterns of responses given by individual participants. So far, in analyzing whether norms change, we’ve tested for significant changes in the normative valence of each action independently of the other actions (e.g. Table 2), but in doing so, we are implicitly treating variation at the individual level as measurement error in our estimate of *the* norm. However, individual heterogeneity may stem from the fact that individuals actually have different beliefs about what the norm is in the situation. Thus, what matters for understanding the impact of norms on behavior in a heterogeneous population is characterizing heterogeneity in beliefs about the norm.

Latent transition analysis (LTA) is a mixture modeling approach^11^ that extends latent class analysis (LCA) to include panel data. LCA identifies unobservable (latent) subgroups (aka classes) from observed response patterns. This approach expresses a multivariate distribution as a composite of a finite number of component distributions, each representing a latent class. LTA is an extension of LCA and uses longitudinal data to identify the degree of movement between the classes over time.

For this reason, LTA is well-suited to uncover the extent and nature of heterogeneity in the norms that participants bring with them and to subsequently test for their stability over time. We thus conduct a series of LTAs, in which we model profiles of normative responses as a function of latent class variables, where each class represents a different norm. Specifically, we identify latent class membership in each wave from the 11 normative valences reported for the dictator game (the ratings of each action from “Give 0” to “Give 10” on our 6-points Likert scale).

Our model restricts the set of classes to be constant across the two time periods and identifies the set of classes that best fit the data, including random intercepts for each participant to control for time-invariant unobservable characteristics (Muthén & Asparouhov, 2022). The key assumptions of the model are conditional independence, i.e. that after controlling for random effects and class membership, the reported normative valences are independent, and measurement invariance, i.e. that the set of latent classes are the same at both time periods (Nylund-Gibson et al., 2022).^12^

Under the model, each latent class can be summarized via an implied probability distribution over the possible responses in the Krupka-Weber norm elicitation task for each of the eleven actions. Comparing model fit statistics (e.g. AIC, BIC), we determined that the best fitting model includes 5 latent classes, which we describe in detail below before examining individual transitions between them.

One important consideration in latent class modelling is the need to distinguish heterogeneity from noise. AIC, BIC, and Adjusted BIC penalize the addition of parameters to the model and trade that off against the improvement in fit. Thus, the convention in LCA and LTA models is to choose the number of classes that minimizes AIC, BIC, and adjusted BIC. In our case all three statistics favor the 5-class model. When they give conflicting results, the convention is to favor the model with fewer classes. As a robustness check, we also estimated LCA models using the wave 1 and wave 2 data separately. As in the LTA estimates, both AIC and BIC favor a 5-class model. Moreover, the norm profiles estimated from both wave 1 and wave 2 show a striking visual resemblance to one another and to the profiles estimated in the 5-class LTA model.

**Fig. 4:**
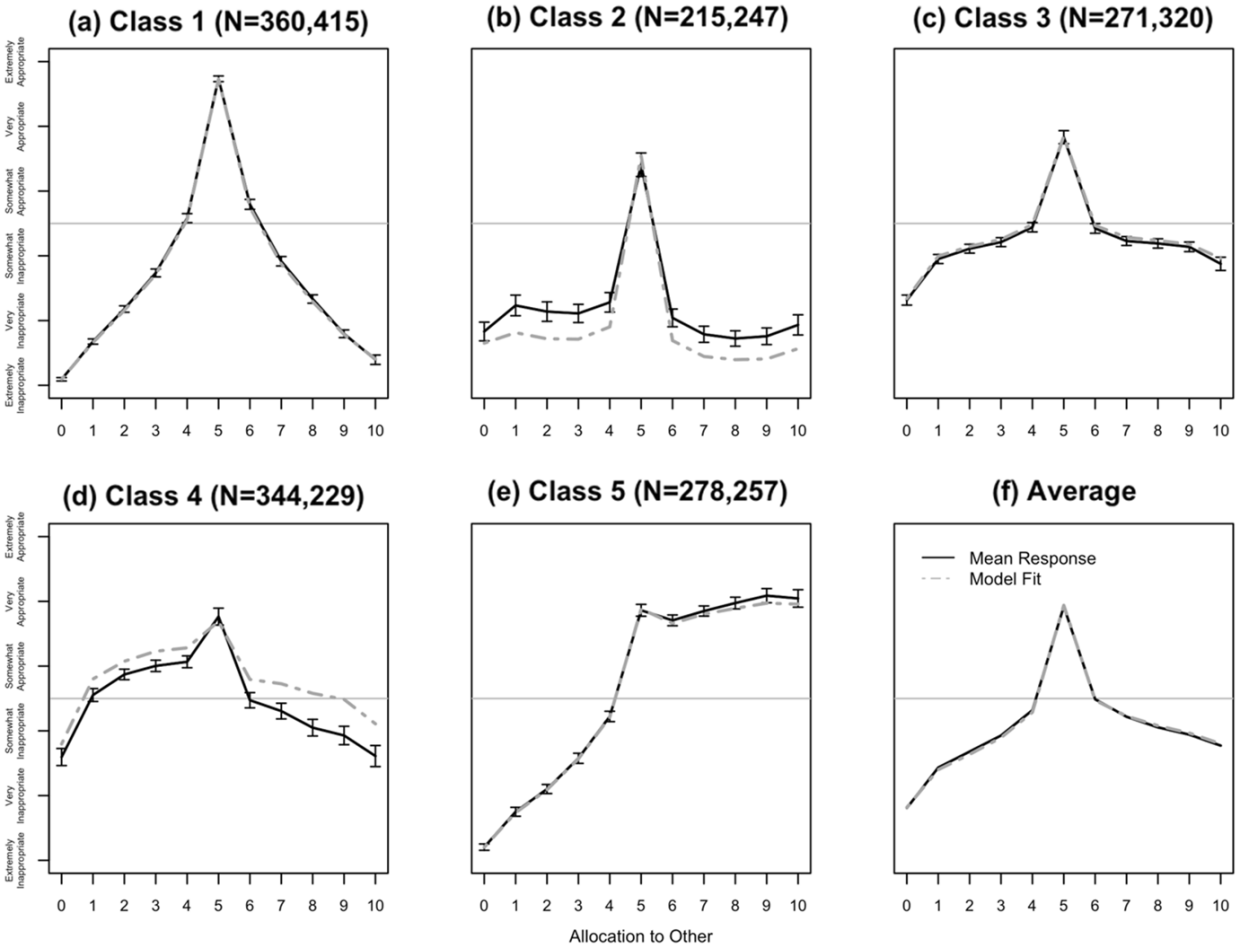
Estimated classes of norms from the latent transition analysis, and mean responses for individuals assigned to each class (± 2SEs). *Note*: The LTA model returns, for each class and each possible dictator action, the estimated probabilities of choosing each normative evaluation (i.e. 6 probabilities that sum to 1). Each grey dashed line plots the mean of those 6 probabilities for each dictator action, in a given class. Intuitively, the grey line captures the expected norm profile for someone in that class. Each solid black line shows the mean reported normative evaluation by subjects assigned to that class in either wave + / − 2 standard errors of the mean. Intuitively, overlap between grey and black shows how well the LTA model classifies the subjects. The solid grey reference line indicates where responses change from approval to disapproval. The title of each panel indicates how many subjects were assigned to that class in each wave

Figure 4 shows both the average responses of subjects who were classified into each class and the implied pattern of responses to the Krupka-Weber elicitation task for each estimated latent class, constructed by computing the expected value of the normative valence of each action, with panel (f) showing the average of the five classes. Each class represents a different injunctive norm, and thus the model reveals substantial heterogeneity in normative beliefs, even in a setting as simple as the dictator game. Under a norm-dependent utility model in which people trade off consumption utility and adherence to injunctive norms (see e.g. Kessler & Leider, 2012; Krupka & Weber, 2013; Kimbrough & Vostroknutov, 2016), each distinct norm will result in a different distribution of choices, depending on the norm that the decision-maker follows.

Moreover, the pattern of responses implied by each latent class has an intuitive interpretation that coheres with widely-known ethical theories and with the kinds of normative interpretation that have been offered in the dictator game (c.f. Yaari & Bar-Hillel, 1984 for early examples of these intuitive classes). The best fitting model includes five latent classes corresponding to five sets of normative beliefs that can be interpreted in terms of what respondents view as “appropriate” (e.g. equality vs. generosity) and how they view deviations (e.g. deontological vs. consequentialist). We attempt to characterize the nature of the norm implied by each class in turn:

*Class 1*: *Strongly egalitarian, consequentialist.* Normative evaluations span the entire range from “extremely inappropriate” when keeping the whole endowment or giving the whole endowment away, to “extremely appropriate” when splitting the endowment equally. This norm strongly favors the equal split, but in keeping with consequentialist ethics, deviations from the normatively best outcome are evaluated according to the magnitude of the deviation. Under norm-dependent utility, such a norm would imply choices that become gradually more self-interested as the intrinsic propensity to follow norms decreases.

*Class 2*: *Egalitarian, deontological*. Like the norm implied by Class 1, this norm strongly favors the egalitarian outcome; however, the appropriateness ratings of all ten inegalitarian outcomes are low and virtually indistinguishable from one another. This suggests a deontological view of dictator game decisions, with any deviation from the normatively best outcome seen as equivalently wrong. Such a norm would imply a bimodal distribution of dictator decisions, with participants either giving half or keeping the whole endowment for themselves, with indifference defined by a threshold value of the norm following propensity.

*Class 3*: *Egalitarian, consequentialist*. This norm has a similar shape to Class 1, but views deviations from the egalitarian ideal as somewhat less problematic overall, while still respecting the consequentialist principle that larger deviations are increasingly unacceptable. Such a norm would again imply a distribution of choices ranging from self-interested to egalitarian depending on the propensity to follow norms, but for a given distribution of norm-following propensities, choices would be skewed toward self-interest, compared to choices under the norm in Class 1.

*Class 4*: *Weakly egoistic*. Class 4 reveals a pattern of normative evaluations consistent with egoism, since nearly every allocation (except for keeping the whole pie and giving away the whole pie) is rated as at least somewhat appropriate. The implied distribution of choices is similar to that in Class 3, but further skewed towards self-interest.

*Class 5*: *Generous, consequentialist*. Class 5 reveals a qualitatively different kind of norm, in which appropriateness is essentially monotonically increasing in the amount given to the recipient. This is consistent with a norm of generosity which respects the consequentialist principle that larger deviations from the ideal are worse. Since appropriateness increases much more rapidly for allocations to the left of the egalitarian outcome than for those to the right, the implied distribution of choices in the dictator game for this norm is quite similar to those for Class 1. Only those with the most extreme norm-following propensities would choose to give more than the egalitarian amount, and so choices in the dictator game would not be likely to reveal the existence of this fundamental difference in normative beliefs.

The existence of such heterogeneity raises questions about how to interpret observed choices in the dictator game. Evidence that “context matters” has already shown that, in general, it is not possible to interpret choices in a dictator game as directly revealing stable social preferences defined over payoff distributions. Models of norm-dependent preferences were designed to address this by showing how dictator games might be used to infer the existence and nature of a shared injunctive norm commonly known to be applicable to a given interaction. The simultaneous existence of multiple normative perspectives on the same interaction further complicates this picture. If each participant’s choices depend on their own normative beliefs and those beliefs vary in the population, then observations of choices from a single dictator game become even more difficult to interpret. In fact, over the range of choices between keeping the whole endowment and the egalitarian allocation, even sharply diverging normative perspectives (e.g. egalitarianism vs. generosity) will generally not be revealed in choices.

**Fig. 5:**
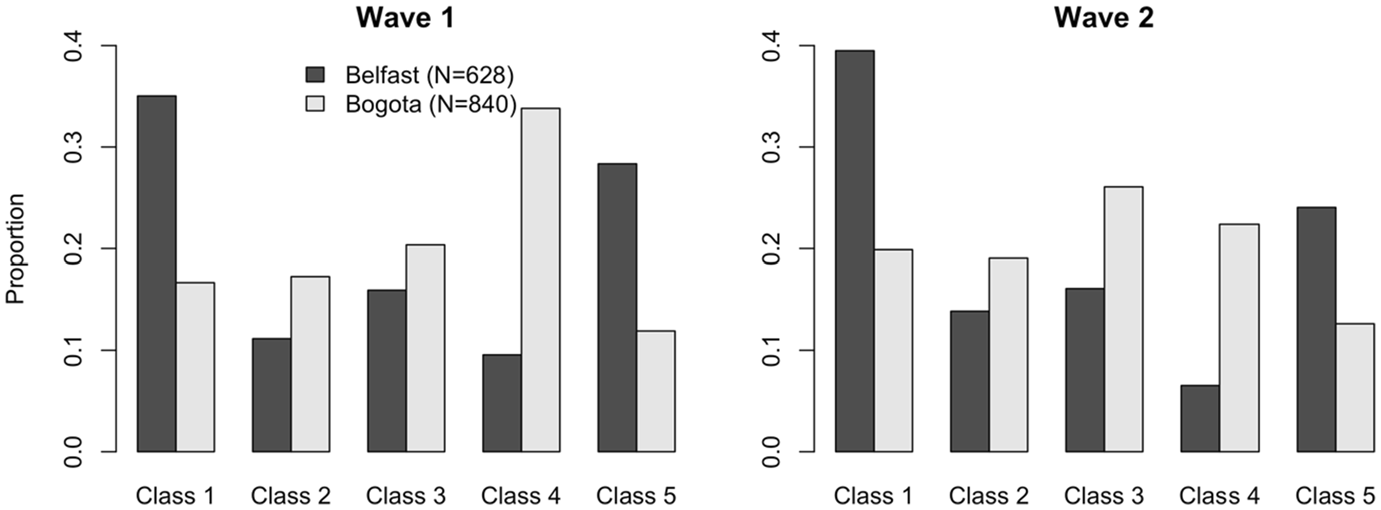
Histogram of best-fitting latent class assignments, by location (Waves 1 and 2)

This interpretive complexity will be further compounded if the distribution of normative beliefs varies across samples. While cross-country heterogeneity is not the focus of this paper (we only have 2 countries to compare), it is worth highlighting that, despite the similarity of *average* normative beliefs in Northern Ireland and Bogotá in our sample, we do see some differences in the relative frequency of the five injunctive norms captured by the latent classes. Figure 5 shows the percentage of subjects whose best-fitting latent class corresponds to each of the five injunctive norms, by location and wave. The data reveal that our Northern Irish subjects are more likely to be classified as Strongly Egalitarian Consequentialists or Generosity Consequentialists than are our subjects from Bogotá. Moreover, our subjects from Bogotá are more likely to be classified as Deontological Egalitarians, Weakly Egalitarian Consequentialists or Egoists. That the averages conceal this difference is perhaps coincidental, but the key implication is that the “same game” need not have the same interpretation in two distinct socio-cultural settings, and thus a focus on averages may ignore important and informative differences.

### Results: evidence of transitions between classes of norms

Having identified 5 classes representing distinct injunctive norms, we now evaluate the stability of individual class assignment over time. The LTA also estimates a transition matrix giving the probability of transitioning between classes across the waves. Table 3a shows the transition probabilities between classes, and Table 3b shows the frequency distribution of types across both waves of the experiment. The strongly egalitarian consequentialist (class 1) and generosity consequentialist (class 5) types exhibit the most stability, with more than half of those classified as those types at T = 1 remaining in the same class at T = 2. No class stands out particularly strongly as an attractor for those who change their classes, though it seems there is a slight tendency for people to transition toward class 3 (and to a slightly lesser extent class 1).

**Table 3:** Transition probabilities and frequency distribution of types (T1 and T2)

T1	T2					T1	T2				
	Class1	Class2	Class3	Class4	Class5		Class1	Class2	Class3	Class4	Class5
(a) Transition probabilities						(b) Class counts					
Class1	0.59	0.11	0.14	0.05	0.11	Class1	217	37	50	17	39
Class2	0.18	0.42	0.20	0.14	0.06	Class2	37	92	44	29	13
Class3	0.21	0.17	0.38	0.14	0.10	Class3	52	47	105	39	28
Class4	0.14	0.13	0.26	0.38	0.09	Class4	48	46	89	129	32
Class5	0.22	0.09	0.12	0.05	0.52	Class5	61	25	32	15	145

To better understand who changes their norms and why, we test whether holding similar normative views to one’s peers at T1 reduces the likelihood of changing one’s normative views by T2 via a linear probability model. Socialization in the time between T1 and T2 may inform subjects that their beliefs differ from their peers’. Thus, we might expect those with distinctive beliefs to change because of the incentives provided by the coordination game. The dependent variable is a dummy variable that takes a value of 1 if a participant’s best fitting latent class changed between T1 and T2 and 0 otherwise. The independent variable of interest is the percent of one’s peers that were in the same latent class as a given participant in T1. As noted above, we define the peer network in three different ways: (1) as the people who a participant nominated as a friend in our social network survey (participants could nominate up to 10 friends in their school year group), (2) as the people in the same classroom as a participant, and (3) as the people in the same school year group as a participant (which is the matching group for the coordination game). The results are reported in Table 4.

**Table 4:** Estimated peer effects on norm stability (i.e. probability of changing latent class assignment), by location

	Friend		Classroom		School	
% in Same latent class at T1	− 0.20***	− 0.45***	− 0.50***	− 1.00***	− 0.64***	− 1.45***
	(0.07)	(0.10)	(0.11)	(0.13)	(0.14)	(0.19)
Bogotá		− 0.08*		− 0.26***		− 0.36***
		(0.05)		(0.06)		(0.07)
Bogotá x % in same latent class at T1		0.46***		1.17***		1.60***
		(0.14)		(0.21)		(0.27)
Constant	0.59***	0.63***	0.66***	0.78***	0.69***	0.88***
[Pr(Change class) | % same = 0)]	(0.02)	(0.04)	(0.03)	(0.04)	(0.04)	(0.05)
N	1115	1121	1121			
Wald test *p* value (Bogotá peer effect > 0)	0.88	0.30	0.46			

On average, those who initially hold normative beliefs that are more similar to their peers are less likely to change their normative beliefs over time, while those who are initially more dissimilar to their peers are more likely to change their beliefs. Note that initial similarity to larger peer groups such as the classroom or the school-year-group shows larger effects than similarity to the self-nominated friend group, despite the fact that the average share of peers who are in the same latent class at T1 is about the same, on average, for each definition of peer group (approx. ¼). This may be explained by the fact that the coordination game was played with the entire school-year-group.

In our setting we should expect to see coordination rates increase over time (due to incentives and socialization over the panel). The transition matrix, along with the regressions in Table 4, provide evidence that speaks to this hypothesis. We find that if a subject is different from their peers, they are more likely to change their beliefs (captured as a change in latent class assignment). Furthermore, similarity to the school-year-group has a larger effect on the likelihood to change beliefs than similarity to the other peer groups, which suggests that respondents are attentive to the incentives (see Table 4). However, the degree of coordination at wave 2 is not substantially higher than that at wave 1. In Northern Ireland, the raw rate at which subjects select the modal normative evaluation over all 11 actions is 0.43 at T1 and 0.44 at T2. In Colombia, the coordination rate is 0.38 at T1 and 0.35 at T2. Thus, we find that subjects change beliefs when dissimilar to their peers, but do not change them in a way that leads them to converge towards a single latent class in each school-year-group. Ultimately, we can predict to some extent who changes, but not how they change.

Table 4 also included specifications with a Bogotá dummy variable and an interaction, to assess whether peer effects vary across contexts. Strikingly, observed increases in consistency over time are almost entirely driven by the Northern Irish sample. Evaluated at the mean observed “% in the same latent class at T1”, we cannot reject the null hypothesis of zero peer effects in Bogotá for any peer group (Wald test that the sum of the coefficients equals zero, *p* values > 0.3), but we can sharply reject the null in Northern Ireland (*p* values < 0.001).

We are reluctant to speculate about the source of these differences. Since we observe norms in only two locations that differ along many socio-cultural dimensions, it is virtually impossible to apportion causality across those dimensions. That said, we note that our samples are drawn from two countries that have been shown to differ substantially on some major social-psychological dimensions. For example, South American countries tend to have relatively more “loose” attitudes toward norms (have weak social norms and a high tolerance of conflicting behavior) compared to Western European countries that tend to be “tight” (have many strong norms and a low tolerance of conflicting behavior) (Gelfand et al., 2011). These observations are consistent with the fact that a slightly higher percentage of subjects in Bogotá were reclassified across waves (55 vs 51%) and the fact that evidence of peer effects on norm change appears to be stronger in Northern Ireland than in Bogotá. Finally, we note that an institutional peculiarity of the Bogotá schools may also have played a role in this finding: the school day is divided into two 4-h blocks due to capacity constraints. One set of students attends in the morning, and another set attends in the afternoon. This probably leads to less interaction between members of the same school-year-group in Bogotá than in Northern Ireland.

## Conclusion

In light of growing evidence that social behavior can be profitably modeled in terms of individual tradeoffs between own consumption utility and normative goals, economists have turned to norm elicitation protocols, such as the coordination game developed by Krupka and Weber (2013), to measure norms because choice data alone is not sufficient for this task. Models of norm-driven behavior tend to assume that norm-motivated agents are influenced by a single, stable, commonly known injunctive norm in each setting. Thus, little work has focused on studying the variation in normative beliefs, on what that variation means, nor on how such variation may be used to tell us about norms or their change over time and across contexts. We show that these basic assumptions about “the” norm do not hold, in the workhorse dictator game. We show how to exploit variation in normative beliefs to extend our understanding of norms in dictator games across two cultural settings and over time. We also show how researchers might use evidence of heterogeneous and changing normative beliefs to study the factors contributing to such changes.

In particular, we use peer networks to predict normative belief change. Previous research has shown that peer networks can be exploited in network interventions (i.e. intervention approaches that purposefully utilize network data within the intervention design). Findings from empirical and simulation-based studies suggest that such approaches could generate behavior change, yet there is little work to date on how to use these data within network interventions to change normative beliefs (Badham et al., 2018, 2019, 2021; Hunter et al., 2019; Valente, 2012). To do so, we use a panel data set on normative beliefs about dictator game giving and a proxy for norm-following propensity from a sample of 1468 participants from two different settings roughly 10 weeks apart.

We first show that a proxy measure capturing norm-following propensity is stable, on average, at the individual level over the sample period. This is consistent with a common assumption in models of norm-dependent utility models that treats norm-following propensity as a fixed, individual-level characteristic. Tate et al., (2022) present a detailed analysis of associations between demographic, personality and cognitive traits and RF task behavior in this sample and find very little evidence that such associations are present among adolescents. A comprehensive multivariate model showed a significant association only with gender, with women putting more balls into the blue bucket than men. This reiterates a finding from Kimbrough and Vostroknutov (2016) who similarly identified gender as the only significant predictor of RF task behavior in their sample of 600 college students. Their evidence suggests that this proxy for norm-following propensity captures a distinctive aspect of decision-making, and our evidence complements this by showing that it reflects a relatively persistent individual-level characteristic.

Moreover, we find that, in aggregate, norm profiles constructed from the *average* normative beliefs for the dictator game are remarkably similar across our two settings and that our targeted age group of respondents, 12 and 13 years old, hold similar normative views, on average, to those documented elsewhere among college age students. However, we also see that a focus on the sample average conceals considerable heterogeneity. To document this heterogeneity, we use latent transition analyses to decompose the aggregate normative belief into latent classes of normative beliefs and to test for, and predict, change in norms over our waves. There are many prior studies showing that differences in behavior *across contexts* are associated with differences in perceived norms (e.g. Krupka & Weber, 2013). Our study suggests that differences in behavior within a *given* context could also be attributable to differences in perceived norms across people within that context.

Our analyses revealed 5 distinct classes representing different norms of dictator giving, each plausibly interpretable as reflecting a well-known ethical perspective. We also find that Northern Ireland and Colombia samples differ, to some extent, in the relative frequency of these 5 classes. The results further show that people transition to different classes from wave 1 to wave 2, and the dissimilarity of peers’ norms to one’s own norms is a significant predictor of the change in norms. So, people who hold similar normative views to their peers are less likely to change their normative views, suggesting that subjects respond to the incentives to coordinate. This effect is only observed in Northern Ireland, which we argue may reflect different patterns of interaction between school-year-group peers across contexts.

We advance research on norms by unpacking the working assumption that there is a single norm profile representing *the* injunctive norm in a given choice context. Normative beliefs regarding the actions one could take are dependent on each other and make the elicitation of an entire profile of normative beliefs imperative. This insight, in turn, can be combined with latent transition analysis to identify latent heterogeneity in beliefs. These analyses, in turn, suggest that observed heterogeneity is not measurement error but rather breaks down into meaningful classes of beliefs: *Strongly egalitarian consequentialist, egalitarian deontological, egalitarian consequentialist, weakly egoistic, and generous consequentialist*. We then use the output of our LTA model to characterize and predict normative belief change.

In short, what we know about norms and what we can test about them is dramatically expanded with these advances. With the modification to our theoretic framework that normative evaluations need to be treated as a profile, and with advances in how we treat variation, we will have much more to say about when a norm is shared in a population, whether it is strong or weak, how and at what moment norm change (at the individual and aggregate level) has taken place.

Our results also have further methodological consequences that need to be worked out. While latent variable models allow us to extract heterogeneous classes of normative beliefs from our data *ex post*, current elicitation techniques are not optimized to reveal such heterogeneity. For one thing, the presence of incentives to coordinate in the Krupka and Weber norm-elicitation protocol means that subjects will tend to report heterogeneous normative beliefs primarily when there is genuine normative uncertainty or unawareness about the most common injunctive norm. Even subjects who recognize that there may be “reasonable disagreement” about whether, say, a generosity norm or an equality norm is most fitting in a given context are forced to report only one norm and face incentives that encourage them to choose the one they believe is shared by the largest proportion of other subjects. This could imply that the heterogeneity we identify is an underestimate of the true heterogeneity. Future work should seek to develop methods that incentive-compatibly elicit beliefs about how many different norms there are, what they look like, and what percent of the population favors each one.

Finally, our findings have relevance not only to our theoretical understandings of norms but also to the practical application of how best to design norms-based policy interventions. Recognition of the importance of individual heterogeneity for the efficacy of such interventions is growing. For example, theorists from network science are now also updating their models to embrace heterogeneities in susceptibility to social influence (Cialdini & Goldstein, 2004), and there is growing evidence for the moderating effects of individual traits like self-efficacy, self-identity and perceived benefits (of behavior change) on the impact of norms-based interventions (e.g. Chung & Rimal, 2016; Murakami et al., 2022; Probst et al., 2020; Rimal et al., 2005; Yun & Silk, 2011). However, there is substantially less work on the interaction between policy interventions and heterogeneous pre-existing norms (for one example, see Pe’er et al., 2019). We suggest this is an important topic for future research.

### Supplementary Information

Below is the link to the electronic supplementary material.

Supplementary file1 (PDF 1313 KB)

## Data Availability

The datasets generated during and/or analyzed during the current study are not publicly available as participants were informed that no-one outside of the research team would have access to the research data when they signed their consent forms. Scripts used to generate our results are available at: https://doi.org/10.7302/w77t-za90. For further information about the study datasets, please contact the authors (Emails: Jennifer.Murray@qub.ac.uk; ruth.hunter@qub.ac.uk).

## Footnotes

1 Economists have studied norms to explain why people are willing to punish others for not cooperating in public goods provision (Fehr and Gaechter 2000), why some communities are able to solve commons problems while others are not (Hardin 1982; Ostrom 2000), why firms adopt particular price setting behaviors (Kahneman et al., 1986) or do not cut wages during periods of high unemployment (Akerlof 1980; Bewley 1998), why countries adopt different redistribution policies (Alesina and Angeletos 2005; Lindbeck et al., 1999), or why teenagers may engage in risky behaviors (Haines and Spear 1996).

2 Injunctive norms, which are shared beliefs about what one ought to do, are distinguished from descriptive norms, which refer to actual patterns of behavior (Cialdini and Trost 1998).

3 It is important to distinguish between the theoretical claim that people have a propensity to follow norms and the existing techniques for eliciting this propensity; see Kimbrough and Vostroknutov (2016, 2018) for a discussion of the relation between this task and the underlying theoretical construct.

4 This was a large public health intervention intended to understand the mechanisms of behavioral change with regards to smoking/vaping behavior among middle-schoolers in two different contexts (Hunter et al., 2020). As List et al. (2023) note, it is not uncommon for studies of policy or behavioral interventions among children to also include evidence regarding the impact of the intervention on non-targeted outcomes, as we do here.

5 We measure peer networks in 3 ways: similarity to self-nominated friends, to classroom peers, and to peers in the whole school year group.

6 In several papers the key assumptions of this protocol—that collectively-recognized social norms create focal points in the matching game – is tested. Burks and Krupka (2012) compare elicited norms with ex-ante identified actual norms and find that the norms elicited using the coordination task track the ex-ante identified norms reliably, while norms elicited without the coordination task and without the matching incentive do not reliably do so. Pushing further on this assumption, Krupka et al. (2017) pair the Krupka and Weber norm-elicitation protocol with (1) an incentivized elicitation of participants’ beliefs about the distribution of actions actually taken by other participants (i.e. they ask raters about their “empirical expectations” as well as their beliefs about the social norm) and (2) a hypothetical question about what they themselves would do in the situation for which they are guessing about the norm. They find that participants’ beliefs about the norm, as measured using the Krupka and Weber norm elicitation protocol, are not primarily driven by their beliefs about others’ likely actions in the games (their empirical expectations), beliefs about what they personally would do in the same situation, nor do the norms ratings substantially differ by a limited set of measured participant characteristics. Fallucchi and Nosenzo (2021) test the vulnerability of the Krupka–Weber method to the presence of alternative salient focal points in two series of experiments with more than 3000 subjects. They find that the method is robust, especially when there are clear normative expectations about what constitutes appropriate behavior.

7 The study of adolescents is important in its own right. Though not the focus of our paper, this subject pool contributes to an important and growing literature studying economic preferences among children. These studies can reveal whether economic behavior develops in patterns over the course of life and it informs theory that seeks to model adolescent choice (eg. such as the decision to take up smoking, which has long term consequences). See Sutter et al. (2019)for a comprehensive review on this topic.

8 While norms for the standard dictator game have turned out to be robust to elicitation with different (primarily WEIRD) populations, we know of little work that elicits them among children in two settings that vary on a number of dimensions. For example, the greater metro area of Bogotá has ± 11 million inhabitants and is the capital city of a upper-middle income country where 31% of the population are under 18 years old. Meanwhile, the entire population of Northern Ireland is about 1.8 million and though it is relatively poor by UK standards, it is well-off by global standards and has only 22% of the population under 18.

9 The ASSIST intervention trains influential peers to deliver anti-smoking information; the Dead Cool intervention employs a more traditional classroom-based pedagogy to teach participants about the influences and risks of smoking.

10 Table A1 in Appendix A presents summary statistics of rule-following and appropriateness ratings in each of the two waves in Northern Ireland and in Bogotá.

11 Mixture modelling approaches are common with behavioral data; see e.g. Conlisk (1989), Harless and Camerer (1994), Harrison and Rutstrom (2009), and Kranton and Sanders (2017).

12 While R and other software packages have native LTA capabilities, they do not have packages that run the Random Intercepts-LTA model that we employ. This implementation exists in the proprietary MPlus software. There are packages in R that allow a user to run MPlus scripts and analyze MPlus output (so long as the user has a copy of MPlus). We have produced a folder containing a script to run our “Random Intercepts-LTA” scripts and produce figures and tables summarizing the revealed latent classes and transition matrices. This folder is available here: https://www.dropbox.com/sh/9dzpi3m0nvuvqy7/AADcmtMMjCxOkvklcCyTdNVka?dl=0
