# Supplementary material for: On the stability of norms and norm-following propensity: a cross-cultural panel study with adolescents
Source: Exp Econ. 2024 May 6;27(2):351–78. doi: 10.1007/s10683-024-09821-5 (PMC11176251; doi:10.1007/s10683-024-09821-5)
Supplement: Supplementary file 1 — Supplementary file1 (PDF 1313 KB) [file 10683_2024_9821_MOESM1_ESM.pdf]

# Appendices for:

## “On the Stability of Norms and Norm-Following Propensity: A Cross-Cultural Panel Study with Adolescents”

Kimbrough, Erik O., Erin L. Krupka, Rajnish Kumar,
Jennifer M. Murray, Abhijit Ramalingam, Sharon Sánchez-Franco,
Olga L. Sarmiento, Frank Kee, and Ruth Hunter

# Appendix A: Additional Analysis and Figures

**Table A1. Baseline and follow-up summary statistics.**

| | **Northern Ireland (N=7)** | | **Bogotá (N=8)** | | **All schools (N=15)** | |
|---|---|---|---|---|---|---|
| | **Baseline** | **Follow-up** | **Baseline** | **Follow-up** | **Baseline** | **Follow-up** |
| Experiment, n | 696 | 684 | 880 | 852 | 1576 | 1536 |
| Survey, n | 701 | 654 | 872 | 846 | 1573 | 1500 |
| **Experiment Part 1 (rule-following task)** | | | | | | |
| Blue bucket (1-50)[a] | | | | | | |
| *Mean (SD)* | 28.8 (19.2) | 29.0 (20.3) | 31.6 (16.9) | 32.7 (17.7) | 30.4 (18.0) | 31.1 (19.0) |
| *Median (IQR)* | 26.0 (11.5 to 50.0) | 27.0 (2.0 to 50.0) | 30.0 (22.0 to 50.0) | 35.0 (23.0 to 50.0) | 28.0 (21.0 to 50.0) | 33.0 (19.5 to 50.0) |
| Yellow bucket (1-50)[a] | | | | | | |
| *Mean (SD)* | 21.2 (19.2) | 21.0 (20.3) | 18.4 (16.9) | 17.3 (17.7) | 19.6 (18.0) | 18.9 (19.0) |
| *Median (IQR)* | 24.0 (0.0 to 38.5) | 23.0 (0.0 to 48.0) | 20.0 (0.0 to 28.0) | 15.0 (0.0 to 27.0) | 22.0 (0.0 to 29.0) | 17.0 (0.0 to 30.5) |
| **Experiment Part 2 (injunctive social norms, dictator game question)[b]** | | | | | | |
| Sit 1.1 (Give £0) | | | | | | |
| *Mean (SD)* | -0.8 (0.4) | -0.8 (0.4) | -0.6 (0.5) | -0.6 (0.5) | -0.7 (0.5) | -0.7 (0.5) |
| *Median (IQR)* | -1.0 (-1.0 to -0.6) | -1.0 (-1.0 to -1.0) | -1.0 (-1.0 to -0.2) | -1.0 (-1.0 to -0.2) | -1.0 (-1.0 to -0.6) | -1.0 (-1.0 to -0.6) |
| *Modal response, n(%)* | 517 (74.4%) | 516 (75.5%) | 447 (50.8%) | 451 (52.9%) | 964 (61.2%) | 967 (63.0%) |
| Sit 1.2 (Give £1) | | | | | | |
| *Mean (SD)* | -0.6 (0.4) | -0.7 (0.4) | -0.2 (0.5) | -0.3 (0.5) | -0.4 (0.5) | -0.5 (0.5) |
| *Median (IQR)* | -0.6 (-1.0 to -0.6) | -0.6 (-1.0 to -0.6) | -0.2 (-0.6 to 0.2) | -0.6 (-0.6 to 0.2) | -0.6 (-0.6 to -0.2) | -0.6 (-1.0 to -0.2) |
| *Modal response, n(%)* | 319 (45.9%) | 322 (47.2%) | 293 (33.3%) | 267 (31.3%) | 612 (38.9%) | 589 (38.4%) |
| Sit 1.3 (Give £2) | | | | | | |
| *Mean (SD)* | -0.5 (0.4) | -0.5 (0.4) | -0.1 (0.5) | -0.3 (0.5) | -0.3 (0.5) | -0.4 (0.5) |
| *Median (IQR)* | -0.6 (-0.6 to -0.2) | -0.6 (-1.0 to -0.2) | -0.2 (-0.6 to 0.2) | -0.2 (-0.6 to 0.2) | -0.2 (-0.6 to 0.2) | -0.6 (-0.6 to -0.2) |
| *Modal response, n(%)* | 305 (43.9%) | 297 (43.5%) | 374 (42.5%) | 263 (30.9%) | 679 (43.1%) | 560 (36.5%) |
| Sit 1.4 (Give £3) | | | | | | |
| *Mean (SD)* | -0.3 (0.4) | -0.4 (0.4) | -0.1 (0.5) | -0.2 (0.5) | -0.2 (0.5) | -0.3 (0.5) |
| *Median (IQR)* | -0.2 (-0.6 to -0.2) | -0.6 (-0.6 to -0.2) | -0.2 (-0.6 to 0.2) | -0.2 (-0.6 to 0.2) | -0.2 (-0.6 to 0.2) | -0.2 (-0.6 to 0.2) |
| *Modal response, n(%)* | 249 (35.9%) | 268 (39.3%) | 290 (33.0%) | 286 (33.6%) | 539 (34.3%) | 554 (36.1%) |
| Sit 1.5 (Give £4) | | | | | | |

| | | | | | | |
|---|---|---|---|---|---|---|
| *Mean (SD)* | -0.1 (0.5) | -0.1 (0.5) | 0.04 (0.5) | -0.1 (0.5) | -0.01 (0.5) | -0.1 (0.5) |
| *Median (IQR)* | -0.2 (-0.2 to 0.2) | -0.2 (-0.2 to 0.2) | 0.2 (-0.2 to 0.2) | -0.2 (-0.2 to 0.2) | 0.2 (-0.2 to 0.2) | -0.2 (-0.2 to 0.2) |
| *Modal response, n(%)* | 281 (40.4%) | 259 (37.9%) | 303 (34.4%) | 269 (31.6%) | 584 (37.1%) | 528 (34.4%) |
| Sit 1.6 (Give £5) | | | | | | |
| *Mean (SD)* | 0.7 (0.5) | 0.7 (0.5) | 0.6 (0.6) | 0.5 (0.6) | 0.6 (0.5) | 0.6 (0.6) |
| *Median (IQR)* | 1.0 (0.6 to 1.0) | 1.0 (0.6 to 1.0) | 1.0 (0.2 to 1.0) | 0.6 (0.2 to 1.0) | 1.0 (0.2 to 1.0) | 1.0 (0.2 to 1.0) |
| *Modal response, n(%)* | 418 (60.1%) | 424 (62.2%) | 445 (50.6%) | 401 (47.1%) | 863 (54.8%) | 825 (53.8%) |
| Sit 1.7 (Give £6) | | | | | | |
| *Mean (SD)* | 0.1 (0.5) | 0.1 (0.6) | -0.01 (0.6) | -0.03 (0.6) | 0.03 (0.6) | 0.01 (0.6) |
| *Median (IQR)* | 0.2 (-0.2 to 0.6) | 0.2 (-0.2 to 0.6) | 0.2 (-0.6 to 0.6) | -0.2 (-0.6 to 0.2) | 0.2 (-0.2 to 0.6) | 0.2 (-0.2 to 0.6) |
| *Modal response, n(%)* | 216 (31.1%) | 186 (27.2%) | 259 (29.4%) | 235 (27.6%) | 475 (30.2%) | 421 (27.4%) |
| Sit 1.8 (Give £7) | | | | | | |
| *Mean (SD)* | -0.1 (0.6) | -0.1 (0.6) | -0.1 (0.6) | -0.1 (0.6) | -0.1 (0.6) | -0.1 (0.6) |
| *Median (IQR)* | -0.2 (-0.6 to 0.6) | -0.2 (-0.6 to 0.2) | -0.2 (-0.6 to 0.2) | -0.2 (-0.6 to 0.2) | -0.2 (-0.6 to 0.2) | -0.2 (-0.6 to 0.2) |
| *Modal response, n(%)* | 188 (27.1%) | 210 (30.7%) | 316 (35.9%) | 265 (31.1%) | 504 (32.0%) | 475 (30.9%) |
| Sit 1.9 (Give £8) | | | | | | |
| *Mean (SD)* | -0.2 (0.7) | -0.2 (0.7) | -0.2 (0.6) | -0.2 (0.6) | -0.2 (0.6) | -0.2 (0.6) |
| *Median (IQR)* | -0.2 (-0.6 to 0.2) | -0.2 (-0.6 to 0.2) | -0.2 (-0.6 to 0.2) | -0.2 (-0.6 to 0.2) | -0.2 (-0.6 to 0.2) | -0.2 (-0.6 to 0.2) |
| *Modal response, n(%)* | 243 (35.0%) | 195 (28.6%) | 253 (28.8%) | 289 (33.9%) | 496 (31.5%) | 484 (31.6%) |
| Sit 1.10 (Give £9) | | | | | | |
| *Mean (SD)* | -0.2 (0.7) | -0.3 (0.7) | -0.2 (0.6) | -0.2 (0.6) | -0.2 (0.7) | -0.3 (0.7) |
| *Median (IQR)* | -0.6 (-1.0 to 0.6) | -0.6 (-1.0 to 0.2) | -0.2 (-0.6 to 0.2) | -0.2 (-0.6 to 0.2) | -0.6 (-0.6 to 0.2) | -0.6 (-1.0 to 0.2) |
| *Modal response, n(%)* | 236 (34.1%) | 255 (37.3%) | 273 (31.0%) | 239 (28.1%) | 509 (32.4%) | 494 (32.2%) |
| Sit 1.11 (Give £10) | | | | | | |
| *Mean (SD)* | -0.3 (0.8) | -0.4 (0.8) | -0.4 (0.7) | -0.3 (0.7) | -0.3 (0.8) | -0.3 (0.8) |
| *Median (IQR)* | -0.6 (-1.0 to 0.6) | -1.0 (-1.0 to 0.2) | -0.6 (-1.0 to 0.2) | -0.6 (-1.0 to 0.2) | -0.6 (-1.0 to 0.2) | -0.6 (-1.0 to 0.2) |
| *Modal response, n(%)* | 327 (47.2%) | 367 (53.7%) | 405 (46.0%) | 340 (39.9%) | 732 (46.5%) | 707 (46.1%) |
| **Survey Big 5 Personality Variables[c]** | | | | | | |
| Openness | | | | | | |
| *Mean (SD)* | 2.4 (0.6) | - | 2.7 (0.7) | - | 2.6 (0.7) | - |
| *Median (IQR)* | 2.4 (2.0 to 2.9) | - | 2.7 (2.2 to 3.2) | - | 2.6 (2.1 to 3.1) | - |
| Extraversion | | | | | | |

| | | | | | | |
|---|---|---|---|---|---|---|
| *Mean (SD)* | 2.6 (0.8) | - | 2.7 (0.7) | - | 2.6 (0.7) | - |
| *Median (IQR)* | 2.6 (2.0 to 3.2) | - | 2.7 (2.2 to 3.2) | - | 2.6 (2.1 to 3.2) | - |
| Agreeableness | | | | | | |
| *Mean (SD)* | 2.5 (0.6) | - | 2.6 (0.7) | - | 2.6 (0.7) | - |
| *Median (IQR)* | 2.5 (2.0 to 3.0) | - | 2.6 (2.1 to 3.1) | - | 2.5 (2.0 to 3.0) | - |
| Conscientiousness | | | | | | |
| *Mean (SD)* | 2.3 (0.7) | - | 2.4 (0.6) | - | 2.4 (0.7) | - |
| *Median (IQR)* | 2.1 (1.9 to 2.7) | - | 2.3 (2.0 to 2.8) | - | 2.2 (1.9 to 2.8) | - |
| Stability | | | | | | |
| *Mean (SD)* | 1.9 (0.8) | - | 2.1 (0.7) | - | 2.0 (0.7) | - |
| *Median (IQR)* | 1.9 (1.3 to 2.4) | - | 2.0 (1.6 to 2.5) | - | 2.0 (1.5 to 2.5) | - |

[a]Number of balls allocated to the blue (rule-following) or yellow (rule-breaking) buckets.

[b]-1=Extremely socially inappropriate; -0.6=Very socially inappropriate; -0.2=Somewhat socially inappropriate; 0.2=Somewhat socially appropriate; 0.6=Very socially appropriate; 1=Extremely socially appropriate.

[c]Each subscale is the average of 10 items coded 0-4. Higher values represent higher levels of the personality trait.

**Table A2: Change in RF Task and Change in Norms by Country**

| | | Northern Ireland (N = 628) | | | | Bogotá (N = 840) | | | | Diff-in-Diff |
|---|---|---|---|---|---|---|---|---|---|---|
| | Measure | Mean | Std. err. | Confidence Interval | | Mean | Std. err. | Confidence Interval | | Mean |
| Norm Following | RF Task | 0.00 | 0.02 | -0.04 | 0.04 | 0.02 | 0.01 | -0.01 | 0.05 | 0.02 |
| | Give 0 | 0.00 | 0.02 | -0.03 | 0.04 | 0.00 | 0.02 | -0.05 | 0.04 | 0.00 |
| | Give 1 | -0.07*** | 0.02 | -0.11 | -0.03 | -0.10*** | 0.02 | -0.14 | -0.05 | 0.03 |
| | Give 2 | -0.06*** | 0.02 | -0.09 | -0.02 | -0.14*** | 0.02 | -0.18 | -0.09 | 0.08 |
| | Give 3 | -0.06*** | 0.02 | -0.10 | -0.02 | -0.08*** | 0.02 | -0.12 | -0.03 | 0.02 |
| | Give 4 | -0.04*** | 0.02 | -0.09 | 0.00 | -0.10*** | 0.02 | -0.14 | -0.05 | 0.06 |
| Appropriateness | Give 5 | 0.01 | 0.02 | -0.04 | 0.05 | -0.07*** | 0.03 | -0.12 | -0.02 | 0.08* |
| | Give 6 | -0.03 | 0.03 | -0.09 | 0.02 | -0.03 | 0.02 | -0.08 | 0.02 | 0.00 |
| | Give 7 | -0.07*** | 0.03 | -0.12 | -0.02 | -0.05 | 0.02 | -0.09 | 0.00 | -0.02 |
| | Give 8 | -0.06 | 0.03 | -0.11 | 0.00 | -0.01 | 0.02 | -0.06 | 0.03 | -0.05 |
| | Give 9 | -0.10 | 0.03 | -0.15 | -0.04 | -0.01 | 0.03 | -0.06 | 0.04 | -0.09 |
| | Give 10 | -0.12 | 0.03 | -0.18 | -0.05 | 0.03 | 0.03 | -0.03 | 0.09 | -0.15*** |

*** $p < 0.01$, ** $p < 0.05$, * $p < 0.10$, Wilcoxon signed-rank or rank-sum tests with Holm-Bonferroni correction to account for multiple comparisons.

## Figure A1: RF Task Histograms by Location

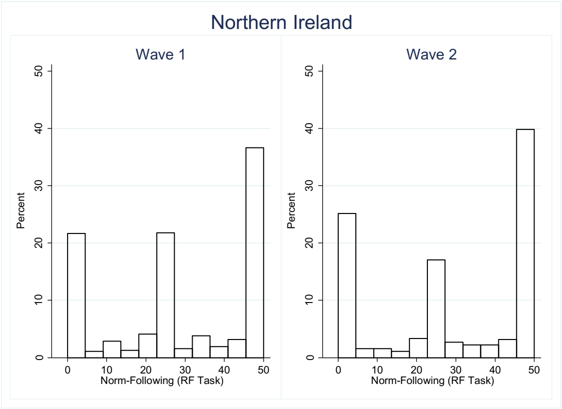


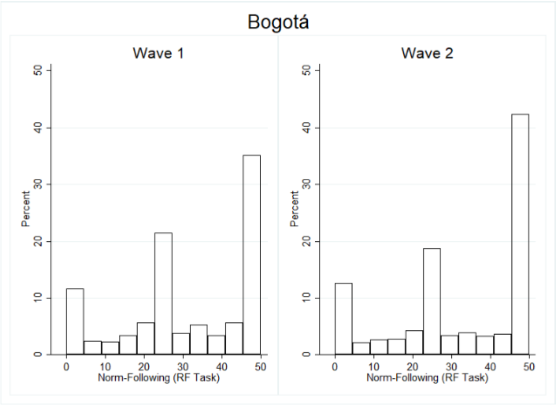


## Figure A2: Norms by Location

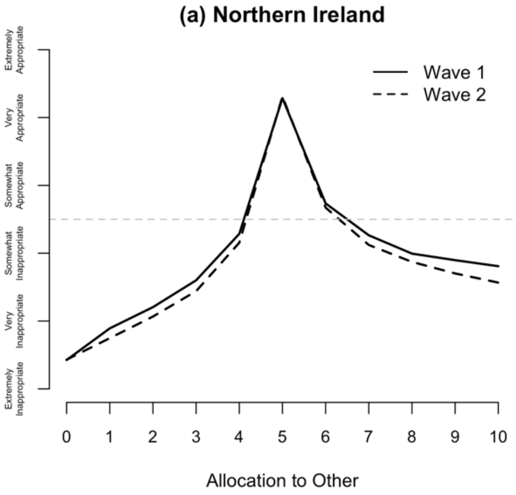


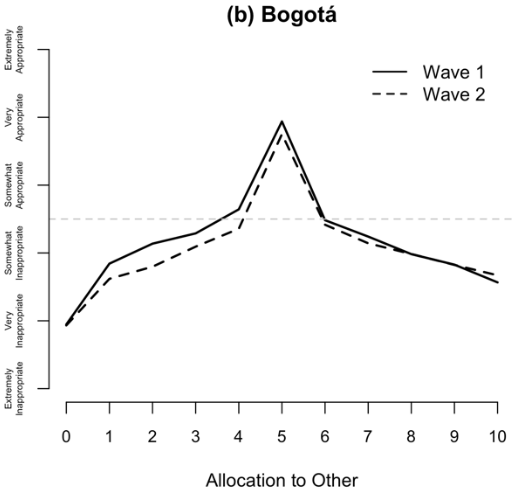

**Figure A3. Individual-level Changes in Norm-Following and Norms, by Location**

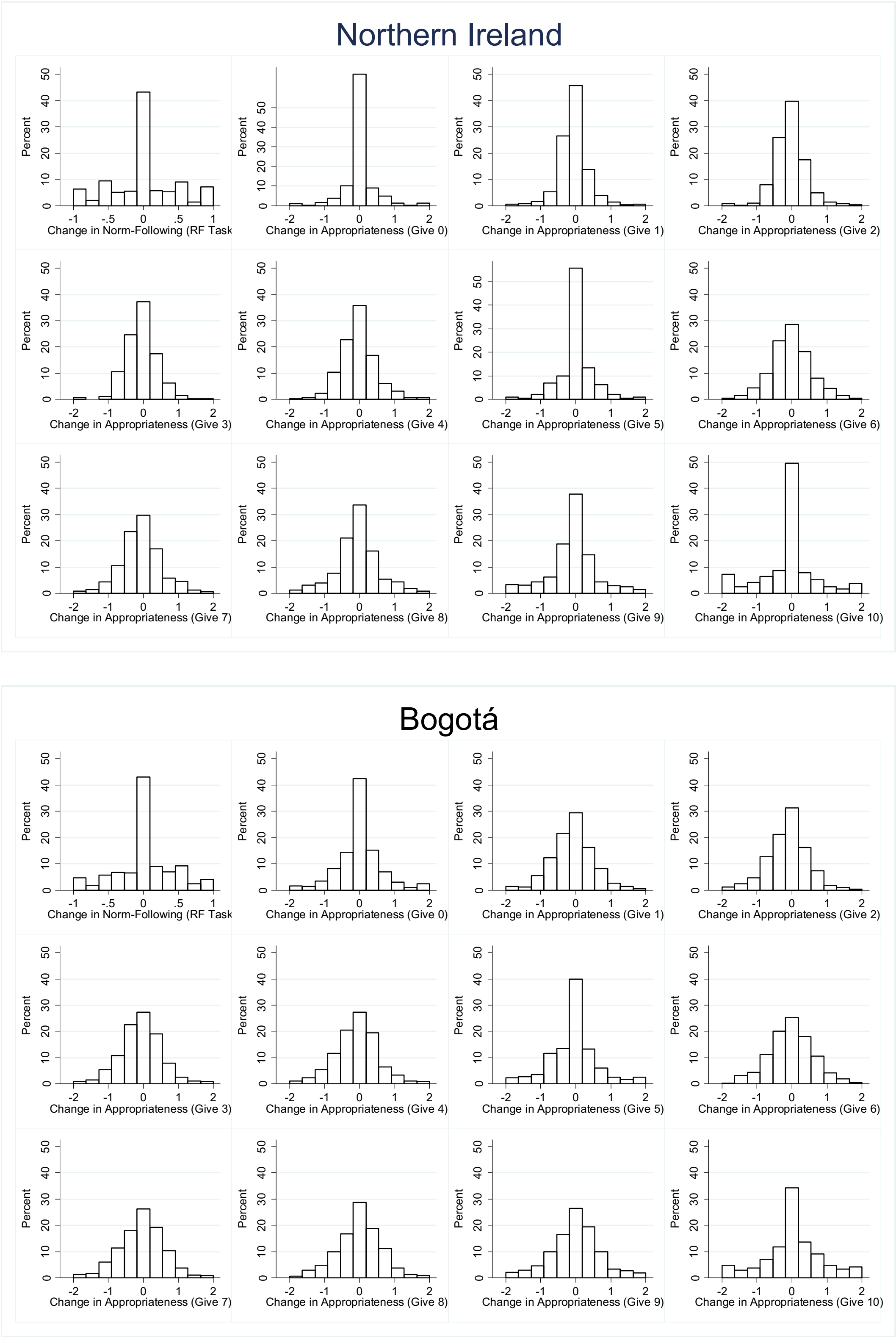

# Appendix B: Experiment Instructions

**English language version of the experimental protocol.**

## Experimental Instructions

### General information

This is a study about decision-making. You will be paid a fee of £5 for taking part, as outlined below. In addition, you may receive some extra money based on your choices and the choices made by others during the study.

If you have any questions during the session, please raise your hand and wait for a researcher to come to you. Please do not talk or try to communicate with other participants during the experiment. It is important that everyone taking part makes his or her own decisions.

This is an on-going study, which has received funding from the UK Medical Research Council to cover all current and future costs. You can be certain that all participants who complete the study will be paid as described in the instructions. If you have any concerns, please contact:

**Dr. Ruth Hunter**
Centre for Public Health/UKCRC Centre of Excellence for Public Health (NI)
School of Medicine, Dentistry and Biomedical Sciences
Institute of Clinical Science B, Royal Victoria Hospital
Grosvenor Road, Belfast, BT 12 6BJ
E-mail: ruth.hunter@qub.ac.uk;
Tel: +44 (0) 28 90978944

### There are four parts to today's study.

### You can earn money in each part.

Your earnings from today will **not** be paid to you today. We will come back to your school at the end of the program in ten weeks' time. At that time, we would like you to participate in another study. There will be four parts to that study, and you can earn money in each part of that study too.

After you have participated in the study at the end of the program we will determine for each part whether you receive earnings from today or from the study at the end of the program. For each part, we will toss a coin to determine this. We will record your choices in both today's study and the study

at the end of the program. You will be able to review your choices from both experiments when you learn your payment, if you wish.

# Part 1

In Part 1 of this study, you will decide how to allocate 50 balls between two buckets. Your task is to put each of the balls, one-by-one, into one of the two buckets: the blue bucket or the yellow bucket. The balls will appear to the left-hand side of your screen, and you can allocate each ball by clicking and dragging it to the bucket of your choice. For each ball you put in the blue bucket, you will receive 5 pence, and for each ball you put in the yellow bucket, you will receive 10 pence.

The rule is to put the balls in the blue bucket.

Once the experiment begins, you will have 5 minutes to put the balls into the buckets. When you are finished, please click on the next button and wait quietly for further instructions from the experimenter. Any balls that have not been placed in a bucket at the end of the 5 minutes are worth nothing. Your earnings from Part 1 will be based on your decisions: it is the sum of earnings from the blue and yellow buckets.

This is the end of the instructions for Part 1. If you have any questions, please raise your hand and a researcher will answer them privately. Otherwise, please wait quietly until all of your classmates are ready and click on the next button to begin the experiment.

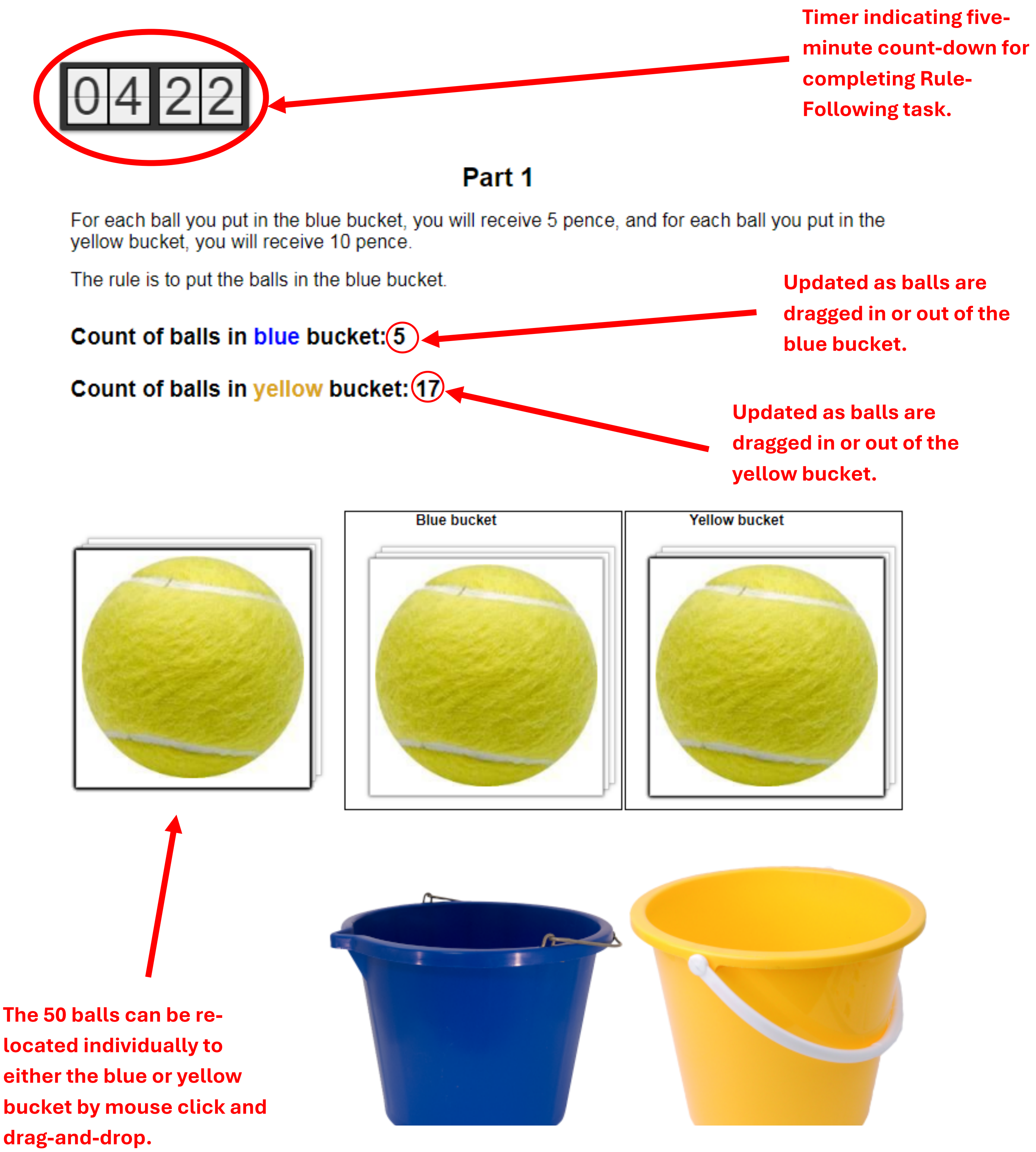


**N.B. Participants were randomized to this version of the experiment or to a version that had the buckets in reverse order to overcome any potential bias due to positioning of buckets.**

# Part 2

On the following screens, you will read descriptions of a series of situations. These descriptions correspond to situations in which one person must make a decision or has taken an action. For each situation, you will be given a description of the decision faced or action taken by this person.

After you read the description of the situation, you will be asked to evaluate the decision or action taken. You will be asked to decide whether taking that decision or action would be "socially appropriate" and "consistent with moral or proper social behaviour" or "socially inappropriate" and "inconsistent with moral or proper social behaviour". By socially appropriate, we mean behaviour that most people in your school year group agree is the "correct" or "ethical" thing to do. Another way to think about what we mean is that if the person in the situation were to select a socially inappropriate choice, then someone else in your school year group might be angry with that person for doing so.

In each of your responses, we would like you to answer as truthfully as possible, based on your opinions of what constitutes socially appropriate or socially inappropriate behaviour.

To give you an idea of how the experiment will proceed, we will go through an example and show you how you will indicate your responses. On the next screen you will see an example of a situation.

# Part 2

## Example Situation

A person is at a local coffee shop near school. While there, the person notices that someone has left a wallet at one of the tables. The person must decide what to do. This person has four possible choices: take the wallet, ask others nearby if the wallet belongs to them, leave the wallet where it is, or give the wallet to the shop manager. The person can choose one of these four options.

*The table below presents a list of the possible choices available to this person. For each of the choices, you will be asked to indicate whether you believe choosing that option is extremely socially inappropriate, very socially inappropriate, somewhat socially inappropriate, somewhat socially appropriate, very socially appropriate, or extremely socially appropriate. To indicate your response, you would select the corresponding option.*

**The person's choice...**

| | Extremely socially inappropriate | Very socially inappropriate | Somewhat socially inappropriate | Somewhat socially appropriate | Very socially appropriate | Extremely socially appropriate |
|---|---|---|---|---|---|---|
| Take the wallet | ○ | ○ | ○ | ○ | ○ | ○ |
| Ask others nearby if the wallet belongs to them | ○ | ○ | ○ | ○ | ○ | ○ |
| Leave the wallet where it is | ○ | ○ | ○ | ○ | ○ | ○ |
| Give the wallet to the shop manager | ○ | ○ | ○ | ○ | ○ | ○ |

**Please make sure that you have placed one tick in each row.**

If this were one of the situations for this study, you would consider each of the possible choices above and, for that choice, indicate the extent to which you believe taking that action would be socially appropriate" and "consistent with moral or proper social behaviour" or "socially inappropriate" and "inconsistent with moral or proper social behaviour". Recall that by socially appropriate we mean behaviour that most people agree is the "correct" or "ethical" thing to do.

# Part 2

For example, suppose you thought that taking the wallet was *extremely socially inappropriate*, asking others nearby if the wallet belongs to them was *somewhat socially appropriate*, leaving the wallet where it is was *somewhat socially inappropriate*, and giving the wallet to the shop manager was *extremely socially appropriate*. Then you would indicate your responses as follows:

**The person's choice...**

| | Extremely socially inappropriate | Very socially inappropriate | Somewhat socially inappropriate | Somewhat socially appropriate | Very socially appropriate | Extremely socially appropriate |
|---|---|---|---|---|---|---|
| Take the wallet | ● | ○ | ○ | ○ | ○ | ○ |
| Ask others nearby if the wallet belongs to them | ○ | ○ | ○ | ● | ○ | ○ |
| Leave the wallet where it is | ○ | ○ | ● | ○ | ○ | ○ |
| Give the wallet to the shop manager | ○ | ○ | ○ | ○ | ○ | ● |

*Are there any questions about this example situation or about how to indicate your responses? On the following pages, there are several situations, all dealing with decisions that another person might have to make.*

*You will indicate your appropriateness rating by selecting the corresponding option.*

*At the end of the experiment today, we will randomly select one of the situations. For this situation, we will also randomly select one of the possible choices that Individual A could make. Thus, we will select both a situation and one possible choice at random. For the choice selected, we will find out which response was selected by most people in your school year group today.*

*If you give the same response as that most frequently given by other people in your school year group, then your earning from Part 2 will be £10. This amount will be paid to you, in cash, at the conclusion of the study in ten weeks. For instance, if we were to select the example situation above and the possible choice "Leave the wallet where it is", and if your response had been "somewhat socially inappropriate", then your earning from Part 2 would be £10, if this was the response selected by most other people in your school year group today. Otherwise your earning from Part 2 would be £0.*

*You are now going to complete some similar questions to this example on your own. You can go at your own pace.*

***If you have any questions from this point on, please raise your hand and wait for the researcher to come to you.***

# Part 2

## Situation 1

Consider two hypothetical individuals from your school year group – Individual A and Individual B. Suppose that Individual A is randomly paired with another person in your school year group, Individual B in an experiment. The pairing is anonymous, meaning that neither individual will ever know the identity of the other individual with whom he or she is paired.

In this hypothetical experiment, Individual A will make a choice, the researcher will record this choice, and then both individuals will be informed of the choice and paid money based on the choice made by Individual A, as well as a small participation fee. Suppose that neither individual will receive any other money for participating in the experiment.

In each pair, Individual A will receive £10. Individual A will then have the opportunity to give any amount of his or her £10 to Individual B. That is, Individual A can give any of the £10 he or she receives to Individual B. For instance, Individual A may decide to give £0 to Individual B and keep £10 for him or herself. Or Individual A may decide to give £10 to Individual B and keep £0 for him or herself. Individual A may also choose to give any other amount between £0 and £10 to Individual B. This choice will determine how much money each will receive, privately and in cash, at the end of the experiment.

*The table below gives a list of the possible choices available to Individual A. For each of the choices, please indicate whether you believe choosing that option is extremely socially inappropriate, very socially inappropriate, somewhat socially inappropriate, somewhat socially appropriate, very socially appropriate, or extremely socially appropriate. To indicate your response, please select the corresponding option.*

**Remember that you will earn money (£10) if your response to a randomly selected question is the same as the most common response provided in your school year group today.**

| | Extremely socially inappropriate | Very socially inappropriate | Somewhat socially inappropriate | Somewhat socially appropriate | Very socially appropriate | Extremely socially appropriate |
|---|---|---|---|---|---|---|
| Give £0 to Individual B (Individual A gets £10, Individual B gets £0) | ○ | ○ | ○ | ○ | ○ | ○ |
| Give £1 to Individual B (Individual A gets £9, Individual B gets £1) | ○ | ○ | ○ | ○ | ○ | ○ |
| Give £2 to Individual B (Individual A gets £8, Individual B gets £2) | ○ | ○ | ○ | ○ | ○ | ○ |
| Give £3 to Individual B (Individual A gets £7, Individual B gets £3) | ○ | ○ | ○ | ○ | ○ | ○ |
| Give £4 to Individual B (Individual A gets £6, Individual B gets £4) | ○ | ○ | ○ | ○ | ○ | ○ |
| Give £5 to Individual B (Individual A gets £5, Individual B gets £5) | ○ | ○ | ○ | ○ | ○ | ○ |
| Give £6 to Individual B (Individual A gets £4, Individual B gets £6) | ○ | ○ | ○ | ○ | ○ | ○ |
| Give £7 to Individual B (Individual A gets | ○ | ○ | ○ | ○ | ○ | ○ |

| | | | | | | |
|---|---|---|---|---|---|---|
| £3, Individual B gets £7) | | | | | | |
| Give £8 to Individual B (Individual A gets £2, Individual B gets £8) | ○ | ○ | ○ | ○ | ○ | ○ |
| Give £9 to Individual B (Individual A gets £1, Individual B gets £9) | ○ | ○ | ○ | ○ | ○ | ○ |
| Give £10 to Individual B (Individual A gets £0, Individual B gets £10) | ○ | ○ | ○ | ○ | ○ | ○ |

**Individual A's choice...**

**If you have any questions, please raise your hand and wait for the experimenter.**

**Social network instructions.**

# MECHANISMS Study Survey 1

Thank you for agreeing to take part in our study. We promise that your answers are confidential. They will not be shown to anyone that you know.

## Instructions

- Please read each question and its options carefully before answering it.
- **If you do not understand a question, please ask for help.**
- Most of the questions can be answered by selecting the answer that applies to you. Sometimes you have to write a number. Sometimes you have to write an answer.
- **Please put up your hand when you have finished.**

**These questions are about your friendship groups in school.**

For these questions, please print the full name of any student you want to suggest. You will be provided with a school year roster to help you. Each student must be in your year group at your school. **Don't name more than ten students.**

Please name **up to ten** of your closest friends in your school year

***Only list those friends who are in your school year****. You do not need to use all the boxes if you do not want to. Do not worry if you are unsure of the spelling. Just try to spell their name as best you can. Please write their full name (i.e. first name and surname), and put a mark (*) beside your best friend's name.*

| | **Name of pupil** | **Form class** |
|---|---|---|
| Name 1 | | |
| Name 2 | | |
| Name 3 | | |
| Name 4 | | |
| Name 5 | | |
| Name 6 | | |
| Name 7 | | |
| Name 8 | | |
| Name 9 | | |
| Name 10 | | |

Please place an X in this box to indicate if your friends are not in your school year.

☐

**Social network data collection.**

Social networks were assessed by asking pupils to name up to ten of their closest friends in their school year group (Dunne et al., 2016; Hunter et al., 2015). Pupils were provided with class rosters and asked to print the full name and school class of their nominated friends. Pupils were instructed not to communicate with other pupils during the data collection. The social networks data was anonymized by matching participants' nominations to class rosters containing each pupil's unique study ID, using the 'agrep' approximate string matching function in R version 3.6.1 (R Core Team, 2019), with maximum distance set to 1. The 'agrep' function automatically matched 90% of the nominations. The remaining 10% of unmatched nominations were independently hand-matched by two researchers, with discussion to resolve disagreements. Throughout this paper, references to 'friendship networks' mean all of the nominated closest friends in the school year group for each focal participant (up to ten).


Dunne, L., Thurston, A., Gildea, A., Kee, F., & Lazenbatt, A. (2016). Protocol: A randomised controlled trial evaluation of Cancer Focus NI's 'Dead Cool' smoking prevention programme in post-primary schools. *International Journal of Educational Research*, *75*, 24–30. https://doi.org/10.1016/j.ijer.2015.06.009

Hunter, R. F., McAneney, H., Davis, M., Tully, M. A., Valente, T. W., & Kee, F. (2015). "Hidden" social networks in behavior change interventions. *American Journal of Public Health*, *105*(3), 513–516.

R Core Team. (2019). *R: A language and environment for statistical computing, R Foundation for Statistical Computing, Vienna, Austria*.
